# Serum metabolite patterns of adipose tissue distribution and body composition subphenotypes

**DOI:** 10.1186/s12944-026-02944-z

**Published:** 2026-04-09

**Authors:** Juliane Maushagen, Elena Grune, Christopher L. Schlett, Lena Sophie Kiefer, Karsten Suhre, Jerzy Adamski, Rui Wang-Sattler, Annette Peters, Fabian Bamberg, Susanne Rospleszcz

**Affiliations:** 1 Department of Diagnostic and Interventional Radiology, Medical Center - University of Freiburg, Faculty of Medicine, University of Freiburg, Freiburg, Germany; 2 Institute of Epidemiology, Helmholtz Munich, Neuherberg, Germany; 3 Department of Diagnostic and Interventional Radiology, Eberhard Karls University of Tuebingen, Tuebingen, Germany; 4 Department of Nuclear Medicine and Clinical Molecular Imaging, Eberhard Karls University of Tuebingen, Tuebingen, Germany; 5 Bioinformatics Core, Weill Cornell Medicine-Qatar, Cornell University, 24144 Education City, Doha Qatar; 6 Department of Physiology and Biophysics, Weill Cornell Medical College, New York, NY 10065 USA; 7 Caryl and Israel Englander Institute for Precision Medicine, New York, NY 10021 USA; 8 Institute of Experimental Genetics, Helmholtz Munich, Neuherberg, Germany; 9 Department of Biochemistry, Yong Loo Lin School of Medicine, National University of Singapore, Singapore, Singapore; 10 Institute of Biochemistry, Faculty of Medicine, University of Ljubljana, Ljubljana, Slovenia; 11 German Center for Diabetes Research (DZD), Munich-Neuherberg, Germany; 12 Institute of Translational Genomics, Helmholtz Munich, Neuherberg, Germany; 13 Chair of Epidemiology, Institute for Medical Information Processing, Biometry, and Epidemiology (IBE), Medical Faculty, Ludwig-Maximilians-Universität (LMU), Munich, Germany; 14 German Center for Cardiovascular Disease Research (DZHK), Munich Heart Alliance, Munich, Germany

**Keywords:** Adipose tissue distribution, Body fat, Metabolomics, MRI, Imaging, Population-based, Epidemiology

## Abstract

**Background:**

Obesity prevalence is increasing globally, accompanied by increases in obesity-related diseases. While obesity is usually defined by BMI, the development of obesity-related disease might be better characterized by specific adipose tissue (AT) distributions or body composition subphenotypes. Serum metabolite patterns reflecting AT distribution could provide insights into potential underlying pathophysiological pathways and the interplay between AT depots. We therefore aim to identify metabolite signatures associated with specific AT depots and body composition subphenotypes.

**Methods:**

Targeted metabolites (Biocrates p180 kit) were measured in fasted serum for *N* = 390 individuals from the population-based KORA-FF4 cohort (42% women, average age 56y). AT was measured by magnetic resonance imaging. Association of *n* = 29 AT depots (visceral (VAT), subcutaneous (SAT), pancreas, bone marrow, skeletal muscle, heart, kidney) and five body composition subphenotypes with 146 metabolites and 40 derived indicators were investigated by linear regressions with confounder adjustment for traditional cardiovascular disease risk factors and life style parameters.

**Results:**

Subphenotypes were associated with 59, and single ATs with 275 metabolites or indicators, with VAT and SAT showing most associations. Compared to subphenotype I (low overall ATs), subphenotype II (average ATs) showed positive associations with diacylglycerophospholipids with differently saturated C32 fatty acid and sphingomyelins. Subphenotype III (high muscle and bone marrow fat) was negatively associated with total lysophosphatidylcholines (lyso-PCs) and total monounsaturated lyso-PCs, while showing a positive association with total long-chain acylcarnitines (C14–C18). Subphenotype IV (high SAT, high VAT and high liver fat) exhibited positive associations with short-chain acylcarnitines, alanine and aromatic amino acids. Subphenotype V (high pancreas fat fraction) was related to arginine and the ratio of ornithine and arginine as surrogate for ornithine synthesis. Three metabolites or indicators (lysoPC C 18.2, total polyunsaturated lyso-PC, phospholipase A2 as ratio of lyso-PC/diacyl- and acylalkylglycerophospholipids) were associated with all subphenotypes. These results were supported by the associations of individual ATs with metabolites or indicators.

**Conclusions:**

ATs, including ectopic fat depots such as pancreas fat, and subphenotypes of body composition show distinct serum metabolite patterns, which can serve as a first step to characterize potential obesity-related pathophysiological pathways.

**Supplementary Information:**

The online version contains supplementary material available at 10.1186/s12944-026-02944-z.

## Background

Obesity is a major public health challenge with a dramatically increasing prevalence. By 2050 more than half of the global adult population is predicted to have at least overweight, if not obesity [1]. Obesity is usually defined by BMI, but the risk of obesity-related cardiometabolic disease is better characterized by specific adipose tissue depots and their distribution [2]. For example, visceral adipose tissue (VAT) shows associations with higher risk of cardiovascular disease (CVD). In comparison, associations for subcutaneous adipose tissue (SAT) are less consistent [3]. Ectopic fat depots such as intrapancreatic fat [2] or skeletal muscle fat are associated with insulin resistance [4] and cardiac adipose tissue is associated with ventricular dysfunction [5]. Moreover, there are well-known sex differences in adipose tissue distribution.

In the same vein, individuals with similar BMI can exhibit different adipose tissue distributions resulting in heterogeneous disease risk [6]. This underscores that the risk for obesity-related diseases might be better captured by specific body composition subphenotypes, determined by adipose tissue distribution [7]. For example, body composition subphenotypes with high liver fat or high overall adipose tissues are associated with higher cardiovascular risk scores [7]. Overall, body composition subphenotypes better represent the interplay between different adipose tissues, which may lead to different diseases, than the single adipose tissue measures. Hence, body composition subphenotypes have the potential to more precisely contribute to the understanding of obesity-related disease progression. This is particularly relevant because (ectopic) adipose tissue accumulation often occurs at several locations, resulting in distinct adipose tissue distributions.

Molecular pathophysiological pathways of obesity-related disease development are not yet fully elucidated. One approach to potentially disentangling the complex relation between body fat and disease development includes metabolomics as intermediate phenotypes from obesity to disease [8]. In this context the evaluation of metabolomics associations with bioelectrical-impedance analysis (BIA) or anthropometric based body composition measures is well established. For example, among other metabolites, sphingomyelins were associated with fat distribution, defined as the ratio of BIA-based trunk to leg fat mass [9]. In a randomized multicenter trial for weight loss, total percentage body fat was associated with lipids, acylcarnitines or amino acids [10]. Furthermore, phospholipids were associated with BMI and suggested to mediate an effect of overweight on diabetes [11]. However, there is a paucity of data on precisely quantified adipose tissue depots, which can be achieved by medical imaging such as magnetic resonance imaging (MRI) or computed tomography (CT). For instance, in two independent cohorts, VAT was associated with acetylglycoproteins, branched‐chain amino acids (isoleucine, leucine, and valine), glutamine (inversely), and serum triglycerides, suggesting them as biomarkers for VAT [12]. Other ectopic fat depots such as pancreas fat were correlated with bile acid and sulfolithocholic acid [13]. However, these findings could not be replicated in the study of the Tübingen Family cohort, as no significant associations between metabolites and ectopic fat were found [14].

Taken together, associations of metabolomics and MRI-based adipose tissue depots can serve as crucial first step to understanding pathophysiological pathways of obesity. Combining body composition subphenotypes with metabolomic profile may further capture the molecular interplay between distinct adipose tissues and their contribution to obesity-related consequences. Also, more detailed metabolomic characterization is necessary by including further body composition measures such as pancreas or cardiac fat in population-based cohort studies. Therefore, in this study, we aim to assess the association of 40 body composition measures and targeted metabolomics. Body composition measures include MRI-based SAT, VAT, ectopic adipose tissues and five derived body composition subphenotypes, as well as BIA-based and anthropometric measures.

## Methods

### Study design

The cross-sectional analysis is based on the population-based cohort study “Cooperative Health Research in the Region of Augsburg” (KORA) using the MRI-subsample of 400 individuals. The MRI study is drawn from the second follow up FF4, *N* = 2279, of the baseline assessment KORA S4, *N* = 4261. Individuals were included in the MRI-substudy, if their age was below 74 years and they had no MRI contraindication or renal impairment, and no known CVD. A comprehensive interview and physical examination were conducted earliest three months before the MRI assessment. The sample was enriched for diabetes and prediabetes cases [15]. The study was performed according to the Declaration of Helsinki, including written informed consent of all participants. The KORA-FF4 study and the -omics measurements therein were approved by the Ethics Committee of the Bavarian Chamber of Physicians (EC No. 06068). The MRI-substudy was additionally approved by the Ethics Committee of Ludwig-Maximilians-University Munich (EC No. 498 − 12).

### Exposure: body composition measures

Our analysis includes a large panel of body composition measures based on MRI-, BIA- and anthropometric- quantification.

For the quantification of adipose tissue, whole body MRI scans were conducted using a 3 Tesla scanner (MAGNETOM, Skyra; Siemens AG Healthcare Sector, Erlangen, Germany) with an 18-channel body coil. Details on the measurements and protocol are described elsewhere [15]. MRI-based body composition measures included abdominal VAT (l), SAT (l) and total adipose tissue (TAT) (l). Furthermore, ectopic adipose tissue was quantified as pancreas fat fraction (%), bone marrow fat fraction (%), skeletal muscle fat fraction (%), cardiac fat measured as volume (ml) and area (cm^2^) and kidney fat fraction (%). A detailed description of MRI-based body composition measures is given in the Supplementary Text (Additional File 1).

Additionally, we calculated the BIA-based body composition measures fat mass index (FMI) [kg/m^2^] and lean mass index (LMI) [kg/m^2^]. Detailed description of BIA-based body composition measures are given in the Supplementary Text (Additional File 1). As anthropometric body composition measures, body mass index (BMI), waist circumference (WC), hip circumference (HC) and waist-to-hip ratio (WHR) were analyzed. BMI was defined as the weight [kg] divided by the square of height [m]; WC measured halfway between the lower rib margin and the iliac crest [cm]; HC measured at the widest protrusion of the gluteal region between the superior border of the iliac crest and crotch [cm]; WHR as WC divided by HC.

### Exposure: body composition subphenotypes

Our group previously derived body composition subphenotypes, based on a panel of MRI-derived adipose tissue [7]. Briefly, subphenotype I is characterized by overall low adipose tissue values; subphenotype II shows average values and slightly increased bone marrow fat fraction; subphenotype III exhibits the highest fat fractions in skeletal muscle and in bone marrow of all subphenotypes; subphenotype IV displays highest liver fat, highest SAT and high VAT values, whereas bone marrow fat fraction and skeletal muscle fat fraction were lower than in subphenotypes II, III and V; subphenotype V represents overall high adipose tissue values with specifically highest pancreas fat fraction (Additional File 1 Table 1).

In total we used 40 body composition measures as exposures, comprising of 34 MRI-based (single adipose tissues and subphenotypes), and 6 BIA-and anthropometric measures.

### Outcome: targeted metabolomics

Serum metabolites were measured using the targeted approach by the AbsoluteIDQ™ p180 kit (BIOCRATES Life Sciences AG, Innsbruck, Austria). Details of metabolites and biochemical groups are given in Additional File 1 Table 2. Out of 188 metabolites, 146 metabolites passed the quality control and were used for analyses. The quality control included a coefficient of variance of < 25%, a limit of detection of < 50% and a non-detectable rate of < 50% [16]. Metabolite-indicators were derived as the sum or ratios of those metabolites that passed quality control (Additional File 1 Table 3). Furthermore, metabolites and indicators were winsorized (95% percentile), logarithmized and standardized per plate to reduce impact of technical errors during measurement [17].

### Clinical characteristics

Participants underwent standardized examinations and were interviewed by trained staff at the study center visit [15]. According to an oral glucose tolerance test or self-report of physicians’ diagnosis, diabetes status was categorized as normoglycemia, prediabetes or diabetes. Individuals were classified as having hypertension if their systolic and diastolic blood pressure were ≥ 140 mmHg and/or ≥ 90 mmHg, respectively, or if they were taking antihypertensive drugs while knowing to have hypertension. Blood lipids were measured after 8 h of fasting using enzymatic, colorimetric Flex assays (Vista, Siemens or Cobas, Roche). Self-reported information was used for alcohol intake (g/day), physical activity (yes or no) and smoking, which was categorized into never, former, or current smoker.

### Statistical analyses

Analyses were conducted on complete cases per exposure variable, leading to samples sizes between 293–390 for the different exposures of body composition (Additional File 1 Fig. 1). To assess the presence of potential selection bias we compared clinical characteristics across these samples by ANOVA or χ^2^-test, with a *p*-value ≤ 0.05 indicating significance.

Continuous variables are described as mean and standard deviation or median and interquartile range. Categorical variables are described as absolute numbers and percentage. In total, 36 body composition measures were analyzed (MRI-based AT, body composition subphenotypes, BIA, anthropometry). For the analysis of categorical subphenotypes, subphenotype I served as the reference category. The MRI-based adipose tissues, BIA and anthropometry measures as exposures were standardized to allow for comparison between the effect estimates. Linear regression models were calculated using two different confounder sets. The base model was adjusted for age, sex and body height as a measure of body size. The full model was additionally adjusted for diabetes status, hypertension, triglycerides, lipid-lowering medication, smoking, alcohol consumption and physical activity. Results are reported as the estimate from the linear regression and 95% confidence interval. To account for multiple testing, p-values were corrected for the false discovery rate (FDR, Benjamini-Hochberg) adjusting for 146 tests. As sensitivity analyses, we excluded individuals taking lipid-lowering medication for the analysis of MRI-based AT and BIA- and anthropometry measures for the full model. Furthermore, we explored sex-specific associations of MRI-based AT and BIA- and anthropometry measures with metabolites adjusting for the full model. Additionally, we tested the robustness of subphenotype-metabolite associations using nonparametric bootstrapped linear regression models (5,000 resamples) adjusted for the full model. Associations were considered significant if the FDR-corrected *p*-value was ≤ 0.05. R version 4.4.0 was used for all analyses.

To evaluate differences in the systemic effect of adipose tissues as represented by the body composition subphenotypes, we calculated pathway analyses. Subphenotype I served as reference to compare subphenotype II-V for enriched pathways. MetaboAnalyst 6.0 [18] was used, considering 80 different pathways for Homo sapiens from the KEGG database. An enrichment analysis was done using Fisher’s exact test was combined with a topology analysis using relative-betweenness centrality. Pathways with an FDR corrected *p*-value ≤ 0.05 were considered statistically significant.

## Results

### Study sample

The largest sample contained *n* = 390 participants with complete BIA and anthropometric data after excluding one participant due to retroactive withdrawal of consent for data usage and 9 participants due to missing metabolite data. Analyses of single adipose tissues and subphenotypes were conducted on samples of 293–375 participants (Additional File 1 Fig. 1). As the analysis samples did not differ significantly in any characteristic, we assume that selection bias is unlikely to have substantially influenced the results (Additional File 2). On average, individuals were 56.4 years old, 42.3% were female and mean BMI was 28.1 (4.9) kg/m^2^ (Table 1 + 2). Diabetes was present in 13.4% of individuals, hypertension in a third of individuals and 11% were taking lipid lowering drugs. Forty percent of individuals were physically active, 20% were currently smoking, and the median alcohol consumption was 8.6 g/day (Table 1).

**Table 1:** Clinical characteristics of the sample

	Overall
n	390
Female sex (%)	165 (42.3)
Age [years]	56.4 (9.2)
Physically inactive (%)	158 (40.5)
Alcohol consumption* [g/day]	8.6 (25.7)
Smoking behavior (%)	
Never smoker	142 (36.4)
Former smoker	170 (43.6)
Current smoker	78 (20.0)
LDL [mg/dl]	139.5 (32.9)
Triglycerides [mg/dl]	131.9 (85.7)
Statin intake (%)	43 (11.0)
Systolic blood pressure [mmHg]	120.6 (16.7)
Diastolic blood pressure [mmHg]	75.3 (10.0)
Hypertension (%)	118 (30.3)
Antihypertensive medication (%)	100 (25.6)
Glycemic status (%)	
Normal	237 (60.8)
Prediabetes	100 (25.6)
Diabetes	53 (13.6)
Antidiabetic medication (%)	31 (7.9)
hsCRP* [mg/l]	1.2 (1.9)
eGFR [ml/min/1.73 m^2^]	89.8 (14.0)

Data are displayed as mean and standard deviation for continuous variables (unless otherwise indicated with an asterisk) and frequency and percentage for categorical variables

^*^Median (IQR); eGFR: estimated glomerular filtration rate; hsCRP: high sensitive C-reative protein

Total adipose tissue (TAT) was on average 12.62 l in the overall sample, whereas VAT and SAT were 4.55 l and 8.10 l, respectively (Table 2). The average cardiac fat volume was 126.2 ml and average pancreas fat fraction 7.7% with small differences among pancreas head, body or tail.

**Table 2:** Body composition measures of the sample based on MRI, BIA and anthropometric quantification

Body composition	Overall
Body fat	
TAT [l]	12.6 (5.5)
VAT [l]	4.6 (2.7)
SAT [l]	8.1 (3.8)
Bone marrow fat	
BMF [%]	54.5 (10.1)
BMF L1 [%]	52.7 (10.3)
BMF L2 [%]	56.3 (10.4)
Cardiac fat	
Total cardiac fat [ml]	126.2 (73.6)
EPCAT [cm^2^]	9.1 (4.7)
PECAT [cm^2^]	30.3 (16.9)
PACAT [cm^2^]	21.2 (13.6)
Pancreas fat	
PF [%]	7.7 (7.0)
PF_cap_ [%]	7.8 (7.3)
PF_cor_ [%]	7.8 (7.2)
PF_cau_ [%]	7.6 (7.0)
Renal fat	
RF [%]	7.7 (3.2)
RSF [%]	63.3 (10.4)
Muscle fat	
SMF pm [%]	7.4 (3.3)
SMF ql [%]	6.8 (3.9)
SMF ar [%]	17.6 (8.3)
SMF ra [%]	15.3 (10.1)
SMF weighted per area [%]	14.2 (6.1)
TOTF pm [%]	7.5 (3.2)
TOTF ql [%]	6.7 (4.2)
TOTF ar [%]	16.6 (8.5)
TOTF ra [%]	16.2 (10.6)
INTRF pm [%]	5.5 (2.0)
INTRF ql [%]	4.2 (1.7)
INTRF ar [%]	8.0 (2. 9)
INTRF ra [%]	7.8 (3.5)
Bioelectrical-impedance measurement	
Fat mass index [kg/m^2^]	9.2 (3.3)
Fat free mass index [kg/m^2^]	18.9 (2.5)
Anthropometric measures	
BMI [kg/m^2^]	28.1 (4.9)
WHR	0.9 (0.1)
Hip circumference [cm]	106. 9 (9.1)
Waist circumference [cm]	98.6 (14.3)

*TAT* total adipose tissue, *VAT* visceral adipose tissue, *SAT* subcutaneous adipose tissue, *BMF L1* bone marrow fat at L1, *BMF L2* bone marrow fat at L2, *HF* heart fat, *EPCAT* epicardial fat, *PECAT* pericardial fat, *PACAT* Paracardial fat, *SMF pm* skeletal muscle fat at psoas major, *SMF ql* skeletal muscle fat at quadratus lumborum, *SMF ar* skeletal muscle fat at autochthonous back muscles, *SMF ra* skeletal muscle fat at rectus abdominis, *SMF* skeletal muscle fat, *TOTF pm* intra- and extracellular fat at psoas major, *TOTF ql* intra- and extracellular fat at quadratus lumborum, *TOTF ar* intra- and extracellular fat at autochthonous back muscles, *TOTF ra* intra- and extracellular fat at rectus abdominis, *INTRF pm* intracellular fat at psoas major, *INTRF ql* intracellular fat at quadratus lumborum, *INTRF ar* intracellular fat at autochthonous back, *INTRF ra* intracellular fat rectus abdominis, *BMI* body mass index, *WHR* waist-to-hip ratio, *RF* renal fat, *RSF* renal sinus fat, *PF* mean pancreas fat, *PFcap* pancreas fat at capus, *PFcor* pancreas fat at corpus, *PFcau* pancreas fat at cauda

### Association of body composition measures with serum metabolites

In the base model, all 35 body composition measures showed associations with metabolites or indicators. The subphenotypes II – V compared to subphenotype I showed 188 associations with metabolites or indicators, the MRI-based measures showed 1038 associations with metabolites or indicators, and BIA-based and anthropometric measures showed 621 associations (Additional File 3).

**Fig. 1:**
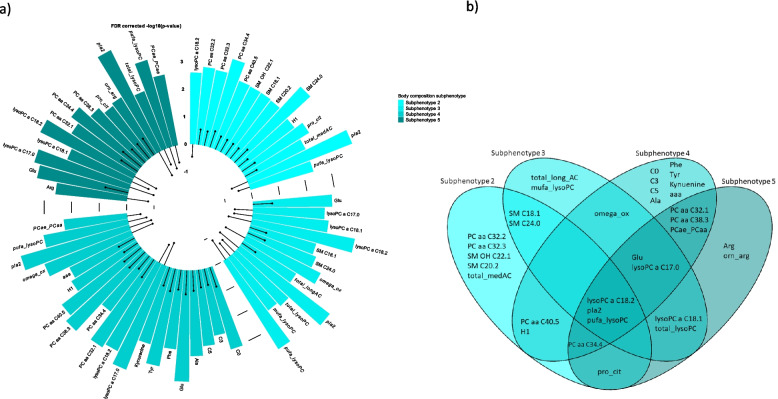
Metabolites associated with subphenotypes. **A** Circular barplot, which shows the associations of subphenotypes with metabolites or indicators of the fully adjusted model (age, sex, body height, smoking, alcohol consumption, physical activity, lipid-lowering medication, diabetes, hypertension and triglycerides). Different subphenotypes are represented by different colors; the height of the bar represents the significance (-log10 p-value) and the dot shows the effect estimate. **B** Venn diagram illustrating the overlap between metabolite that were significantly associated with subphenotypes in the fully adjusted model

**Fig. 2:**
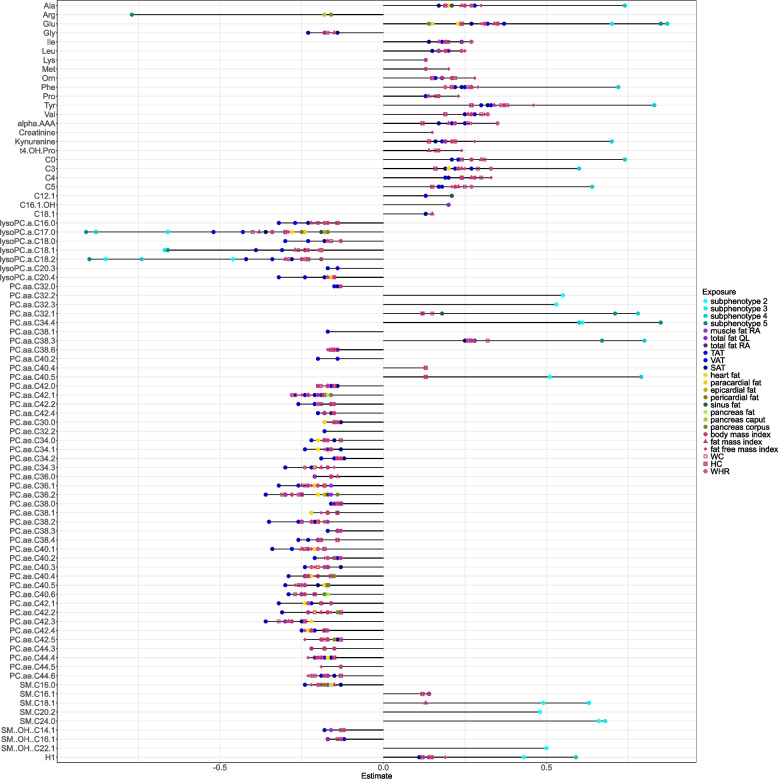
Metabolites associated with body composition measures. Lollipop plot showing the significant associations of body composition measures with metabolites of the fully adjusted model (age, sex, body height, smoking, alcohol consumption, physical activity, lipid-lowering medication, diabetes, hypertension and triglycerides). The x-axis shows the effect estimate and the y-axis significant metabolites (amino acids, biogenic amines, phospholipids, sphingomyelins and sugar). Different shades of light blue indicate subphenotypes II-V and purple, green, blue and yellow show MRI-based adipose tissues. Body composition exposures based on BIA- and anthropometric- measures are shown in red by different shapes. Abbreviations: QL = quadratus lumborum; RA = rectus abdominis; TAT = total adipose tissue, VAT = visceral adipose tissue; SAT = subcutaneous adipose tissue; WC = waist circumference; WHR = waist to hip ratio; HC = hip circumference

In the full model, in total 777 associations remained, of which 60 associations were related to the subphenotypes (Fig. 1), 308 to MRI-based measures and 409 to BIA-based and anthropometric measures (Fig. 2, Additional File 4).

### Metabolite signatures of subphenotypes

Results from the base model are shown in Supplementary Table 3. In the full model, the subphenotypes II-V were associated with 60 metabolites or indicators compared to subphenotype I (Fig. 1). Lyso-PC a C18:2, PlA_2_ (surrogate for phospholipase A_2_) and total polyunsaturated lyso-PCs were negatively associated with all subphenotypes. Additionally, all subphenotypes except for the subphenotype II, showed a positive relation to glutamate and an inverse association with lyso-PC a C17:0. This indicates a potential link to general increase in adipose tissue as subphenotypes II-V show higher adipose tissue than subphenotype I. Subphenotypes IV and V demonstrated overlapping metabolite associations for PC aa C32:1/C38:3 and the ratio of acyl-alkyl- to diacyl-PCs. In comparison, subphenotypes IV and III shared associations with omega oxidation (Fig. 1B). Subphenotype V and III were both associated with lysoPC C 18.1 and total lysoPCs.

In contrast to these overlapping associations, we also found subphenotype specific associations. In detail, subphenotype II was characterized by average amount of adipose tissue, low prevalence of hypertension and large proportion of currently smoking individuals. This subphenotype showed positive associations with medium long-chain acylcarnitines, diacyl-PC with differently saturated C32 fatty acid and sphingomyelins. Bone marrow fat fraction, which was also elevated in subphenotype II, was not significantly associated with any metabolite in single adipose tissue analyses. Subphenotype III was characterized by highest age and high prevalence of hypertension, high skeletal muscle fat fraction and high bone marrow fat fraction. This subphenotype was negatively associated with total monosaturated lyso-PCs while showing a positive association with total long-chain acylcarnitines (C14 – C18). Subphenotype IV shows a high prevalence of hypertension at a comparable young average age (55y) with increased SAT, VAT and high liver fat. This subphenotype showed most associations of all subphenotypes and exhibited positive associations with short-chain acylcarnitines, alanine and aromatic amino acids. In associations of single adipose tissues, SAT and VAT showed similar associations as subphenotype IV and additionally with branched-chain amino acids. Finally, subphenotype V is marked by highest proportions of hypertension, diabetes and strongly increased pancreas fat fraction. This subphenotype was inversely related to arginine levels and positively related to the ratio of ornithine and arginine, a surrogate for ornithine synthesis (Fig. 1). Pancreas fat fraction from the analysis of single adipose tissues was inversely associated with arginine levels and five PCs (Fig. 2, Additional File 1 Fig. 2 + 3). Further findings from the analysis of single adipose tissues showed that cardiac fat volume was associated with (lyso-)PCs (inversely), glutamate and alanine.

### Metabolite patterns of BIA- and anthropometry-based measures

Results from the base model are shown in Additional File 3. In the full model, the BIA- and anthropometric-based body composition measures exhibited 409 associations including most metabolites. We observed most associations for BMI (80), FMI (79) and WC (76) with most metabolites overlapping among these exposures. The directions of associations were consistent within metabolites (Fig. 2, Additional File 1 Fig. 2 + 3). BIA- and anthropometric-based body composition measures were inversely associated with lyso-PCs, PCs, and indicators for total number of lipids, with one exceptionally direct association for PC aa C38.3. Positive associations were found for amino acids, AAA, BCAA, and short-chain acylcarnitines. BIA- and anthropometric-based body composition measures showed a similar metabolomic pattern as MRI-measured TAT, VAT and SAT, indicating that these metabolites are robustly associated with body fat, irrespectively of the type of measure (Fig. 2, Additional File 1 Fig. 2 + 3). However, some associations were not identifiable by BIA- or anthropometric-based measures. For example, we found the association of pancreas fat fraction and arginine only in the single adipose tissue analysis and subphenotype V. In comparison, the global arginine bioavailability ratio (arg/orn + cit) was negatively associated only with anthropometric-based measures (BMI, FMI, WC, WHR, LBM) (Additional File 1 Fig. 3).

Detailed effect estimates, confidence intervals, raw and corrected p-values according to body composition measure can be found in Additional File 4.

### Sensitivity analyses

We assessed the associations of adipose tissue, BIA- and anthropometry measures with metabolites in individuals without lipid-lowering medication intake to test robustness of associations in the full model. The effect estimates did not change substantially compared to the main findings, indicating robust associations (Additional File 1 Fig. 4, Additional File 6). In additional sex-specific analyses we found a similar pattern of associations for men compared to the main findings (568 associations), while women showed fewer associations (143 associations) (Additional File 1 Fig. 5 + 6, Additional File 7). Compared to the main findings we found 84 overlapping associations between men, women and the whole sample while 399 associations overlapped between men and the whole sample (Additional File 1 Fig. 7), indicating that most associations were driven by men in the main analysis. The sex-specific findings warrant further studies assessing sex-specific associations in larger cohorts. The stability analysis of metabolite-subphenotype associations using bootstrapped linear regression adjusted for the full model indicated mostly stable associations. We found 55 significant associations with 51 overlaps with the main analysis and similar effect estimates (Additional File 1 Fig. 8 + Additional File 1 Table 4).

### Pathway analysis of subphenotypes

**Fig. 3:**
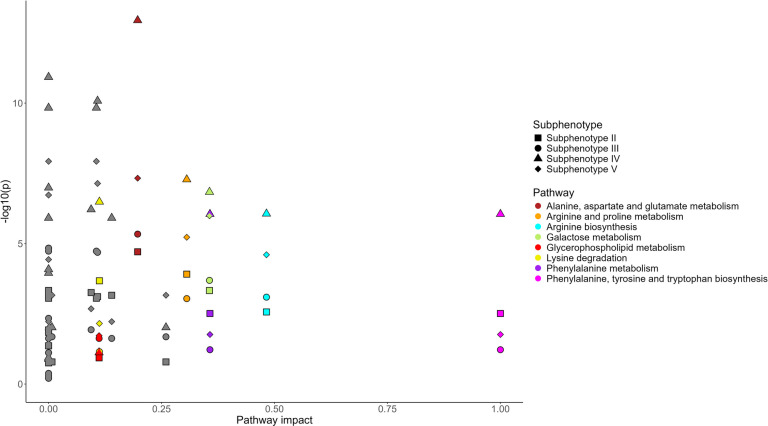
Pathway analysis of subphenotypes. Pathway analysis of subphenotype II-V with subphenotype I as reference based on all metabolites that were significant in the fully adjusted model. The x-axis shows the pathway impact as importance measures and y-axis the negative log10(*p*-value) as enrichment measure. The shape of points indicates the subphenotypes II – V, and the colors selected pathways. Subphenotype IV shows the highest enrichment for most pathways and subphenotype III the lowest enrichment for most pathways

We compared pathway enrichment analysis of subphenotypes II-V, each compared to subphenotype I using metabolites that were significantly associated with any significant metabolite from the full model. Subphenotype IV showed the highest enrichments throughout all significant pathways (Fig. 3, Additional File 5). Subphenotype V showed highest number of significant enriched pathways (Additional File 5). Pathways that were solely enriched in subphenotype V were arachidonic and linolenic acid related pathways. The alanine, aspartate and glutamate metabolism was strongest enriched in subphenotypes II-IV, whereas the most significantly enriched in subphenotype V were the pathways of glyoxylate and dicarboxylate metabolism and porphyrin metabolism. Generally, the pathway profiles of all four subphenotypes were similar only with subphenotype V showing slight differences.

## Discussion

In this sample from a population-based cohort, we investigated the cross-sectional association of a comprehensive panel of body composition measures, including imaging-derived adipose tissue depots, subphenotypes, BIA- and anthropometric-based measures with targeted serum metabolites. Our two main findings are, (1) that metabolites are indicative of increased body size across different body composition measures and (2) that specific adipose tissue distributions, indicated by body composition subphenotypes, show moderately different metabolite profiles.

### Metabolite associations of overall body composition and subphenotypes

We found specific metabolites to be associated with all body composition measures (MRI-based, BIA-based and anthropometric), as well as metabolites that were specific to certain adipose tissues or subphenotypes. Forty-four PCs, *7* lyso-PCs, 18 amino acids and biogenic amines were associated with BIA- and anthropometric-based measures (BMI, WC, FMI, WHR, HC), as well as with all imaging-derived subphenotypes (Fig. 2, Additional File 1 Fig. 2), VAT, SAT, cardiac fat volume, renal fat fraction or skeletal muscle fat fraction at rectus abdominis.

Carbon chain length and degree of saturation of fatty acids in PCs modulates their association with obesity [19], which is in line with our finding that most diacyl-PCs and all acyl-alkyl-PCs showed inverse associations. Only PC aa C38:3 was positively associated with multiple anthropometric body composition markers (Fig. 2, Additional File 1 Fig. 2). PC aa C38:3 has been suggested as a mediating factor for a progression from obesity to type 2 diabetes [11]. In an intervention study on body weight, associations of body fat with similar PCs in both directions is in line with our finding [10]. However, the intervention study found the association only in unadjusted analyses, while our results are fully adjusted. Further to that, in a previous study of the current sample acyl-alkyl-PCs were inversely associated with liver fat, and only PC aa C32:1 showed positive associations in men [17]. In an analysis of 10 cohorts, several metabolite classes – including glycerophospholipids – showed genetic correlations with SAT, VAT, and liver fat [20]. This supports a potential mechanistic role for these metabolites linking obesity and metabolic disease [20].

In our analysis lyso-PC C 17.0 showed most associations with body composition measures including subphenotypes III-V and lyso-PC C 18.0 was inversely associated with every subphenotype. These findings are confirmed in a study of German cohorts [21] and in another study on healthy women lyso-PCs were inversely associated with the ratio of VAT/SAT [22].

There likely is a bi-directional relationship between adipose tissue and circulating (lyso) PCs, with increased lipid peroxidation in obesity resulting in lower levels of PCs [23]. At the same time lipolysis or apoptosis is induced by PCs in specific adipocytes [24]. If (lyso-) PCs get depleted, lipid turnover is disrupted which results in local inflammation [25] or insulin resistance [26]. Thus, their association extends to other cardiometabolic health states; for example, in another analysis of the current sample, higher PCs were associated with favorable cardiac parameters [16]. Moreover, a protective effect of acyl-alkyl-PCs was found for metabolic syndrome [27] and dietary intake of PCs was associated with lower risk for type 2 diabetes in men [28].

In our analysis, nine acyl-alkyl-PCs were additionally inversely associated with epicardial fat volume and pancreas fat fraction. These adipose tissue depots have not been extensively studied in population-based research, but are gaining interest because of potential relevance in heart failure pathogenesis [29] and diabetes development [30]. In the MESA cohort negative associations of PCs and cardiac fat are in line with our results [31]. In comparison, smaller studies including women with obesity [32] or a pedigree-based sample of Mexican Americans [33] showed positive associations. Taken together, metabolomics shows strong potential to unlock the metabolic interplay of cardiac adipose tissue and CVD. However, longitudinal studies with replication samples are warranted to confirm findings and elucidate on the effect direction.

Regarding amino acids, we found that glutamate was positively associated with most body composition measures, including all subphenotypes, cardiac fat volume and pancreas fat fraction. Associations of serum glutamate with obesity markers have been shown previously [34]. Additionally, we found that aromatic (AAA) and branched-chain (BCAA) amino acids were associated with increased VAT, SAT and anthropometric-based body composition measures. This is in line with previous findings from this sample where BCAA were linked to higher hepatic fat [17]. Previous research from another population-based sample has corroborated an association with VAT and SAT [35]. A dysfunctional catabolism of BCAA is also associated with metabolic diseases such as insulin resistance [36] and predicts future type 2 diabetes [20]. Hence, BCAA might represent one potential molecular link between increased adipose tissue and metabolic diseases.

### Subphenotype-specific metabolite associations

Imaging-derived subphenotypes reflect a holistic interplay between different adipose tissue depots, offering a more comprehensive picture of body fat distribution. Compared to traditional anthropometric measures, subphenotypes better capture clinically relevant patterns of fat distribution [7]. In contrast, other population-based cohorts or intervention studies have identified associations of amino acids [37] or lipids [10] with mostly anthropometric-based body composition measures which are mainly in line with our results. However, metabolite profiles of imaging-derived subphenotypes offer the opportunity to provide insights about molecular connection between ectopic fat depots and potential health outcomes as a first step. Overall, we found only few metabolites that were specific to distinct subphenotypes.

For example, short-chain acylcarnitines (C0, C3, C5) were specific to subphenotype IV together with phenylalanine, tyrosine and the sum of AAA and alanine. C5 has previously been associated with VAT in individuals with high VAT [38], supporting our results. In contrast, the sum of long chain acylcarnitines was specific to subphenotype III. For subphenotypes III and IV we observed inverse associations with relative omega-oxidation (ratio of dicarboxylic acylcarnitines to total acylcarnitines). This likely reflects an increase of the total acylcarnitines driven by increased long-chain acylcarnitines in subphenotype III and increased short-chain acylcarnitines in subphenotype IV. Both long- and short-chain acylcarnitines are associated with obesity, type 2 diabetes and increased blood pressure [39] or unfavorable left ventricular parameters [16]. Subphenotype III and IV show indeed high proportions of diabetes or hypertension and are associated with increased CVD risk according to SCORE2 compared to subphenotype II [7].

For subphenotype V, we found a negative association with arginine and a positive association with the ratio of ornithine to arginine reflecting ornithine synthesis. Arginine was also negatively associated with pancreas fat fraction, which is highest in subphenotype V. Only few studies have assessed pancreas fat and metabolomics. Lind et al. found pancreas fat to be associated with 5-hydroxylsine and taurodeoxycholate [38], another study found no associations in individuals with prediabetes [14] and a third cohort study found no significant metabolites after adjusting for age [13]. Although our results are not yet confirmed by other cohort studies, the inverse relation is plausible, considering that arginine stimulates lipolysis in adipocytes. However, this effect seems to depend on the tissue and stage of adipocyte differentiation [40]. Increased pancreas fat is a risk factor for pancreatic diseases [41] and in pancreatitis in rodents, arginine and arginase exacerbate pancreatitis [42]. The expression of arginase 2 –an enzyme that converts arginine to ornithine while producing nitric oxide (NO)– was increased in human cells and mice with obesity suggesting that arginase 2 potentially links pancreas fat to pancreas cancer [43]. Hence, a potential explanation for the inverse association with arginine in our analysis could be that arginase activity is increased in adipose tissue. This can be supported by the positive associations observed for ornithine/arginine ratio and ornithine in our analysis. We therefore hypothesize that arginase activity might already be elevated during the stage of adipose tissue accumulation preceding the onset of overt pancreas diseases. However, since our analysis does not include circulating NO or urea measurements, this hypothesis remains speculative. Additionally, our findings are novel and based on cross-sectional data. Therefore, our findings do not allow for causal inference or determination of effect direction. Replication of associations is needed in larger and longitudinal studies.

The pathway analyses of subphenotypes II-V compared to subphenotype I showed similar pathways to be enriched among subphenotypes. Strikingly, subphenotype IV showed highest enrichment in most pathways and subphenotype III showed mostly lowest enrichment of pathways. We hypothesize that these differences in pathways might be due to the high proportion of age-related metabolic diseases at a comparably younger average age in subphenotype IV compared to subphenotype III.

### Limitations

Our findings have to be interpreted in light of their limitations. While the deeply phenotyped sample, combining targeted metabolomics with a large panel of body composition parameters, including BIA and precise MRI-based quantification, is a major strength of our study, our data were observational, cross-sectional and based on a small sample, limiting statistical power. For example, it is possible that we failed to see consistent sex-specific associations due to the small sample size. The AbsoluteIDQ™ p180 kit (BIOCRATES Life Sciences AG, Innsbruck, Austria) shows certain advantages, for example, that the interlaboratory reproducibility was extensively evaluated [44]. However, it is important to consider that the targeted kit only represents a small proportion of the whole metabolome. Particularly the panel of lipid is limited compared to non-targeted, high-resolution mass spectrometry which means that we might miss important lipid-signaling pathways, that could serve to explain other differences between subphenotypes. Furthermore, our results need to be replicated in independent studies before robust conclusions can be drawn. Therefore, the identified metabolic signatures remain preliminary until external and larger cohort studies validate findings.

## Conclusion

Adipose tissues and body composition measures show distinct associations with metabolites, particularly for ectopic fat depots such as pancreas fat. Body composition subphenotypes based on adipose tissue distribution show moderately distinct metabolite associations. Our results can serve as first steps to elucidate the underlying pathophysiological paths of obesity-related consequences, considering the interplay between adipose tissues.

## Supplementary Information

Additional file 1. Supplemental text, figures and tables.

Additional file 2. Comparison of analysis samples.

Additional file 3. Results for the base model.

Additional file 4. Results for the full model.

Additional file 5. Results for the pathway analysis.

Additional file 6. Results from the sensitivity analysis excluding individuals taking lipid-lowering medication adjusted for the full model.

Additional file 7. Results from the sex-stratified sensitivity analyses adjusted for the full model.

## Data Availability

Data are available upon request by means of a project agreement from KORA. Requests should be sent to kora.passt@helmholtz-munich.de and are subject to approval by the KORA Board. Analysis codes are available from the authors upon reasonable request.

## Abbreviations

AAA: Aromatic amino acids
acyl-alkyl-PC: Acylalkylphosphatidylcholines
ar: Autochthonous back muscles
BCAA: Branched-chain amino acids
BIA: Bioelectrical-impedance analysis
BMAT L1/L2: Bone marrow adipose tissue at L1/L2
BMI: Body mass index
CT: Computer tomography
diacyl-PC: Diacylphosphatidylcholines
EPCAT: Epicardial fat
FMI: Fat mass index
HC: Hip circumference
hsCRP: High sensitive C-reactive protein
INTRF: Intracellular fat fraction
KORA: Cooperative Health Research in the Region of Augsburg
LMI: Lean mass index
lyso-PC: Lysophosphatidylcholines
MRI: Magnetic resonance imaging
PACAT: Paracardial fat
PECAT: Pericardial fat
PFF: Mean pancreas fat fraction
PFFcap: Pancreas fat fraction at capus
PFFcau: Pancreas fat fraction at cauda
PFFcor: Pancreas fat fraction at corpus
pm: Psoas major
ql: Quadratus lumborum
ra: Rectus abdominis
RFF: Renal fat fraction
RSFF: Renal sinus fat fraction
SAT: Subcutaneous adipose tissue
SM: Sphingomyelins
SMFF: Skeletal muscle fat fraction
TAT: Total adipose tissue
TOTFF: Intra- and extracellular fat
VAT: Visceral adipose tissue
WC: Waist circumference
WHR: Waist to hip ratio
