# Supplementary material for: Serum metabolite patterns of adipose tissue distribution and body composition subphenotypes
Source: Lipids Health Dis. 2026 Apr 9;25:109. doi: 10.1186/s12944-026-02944-z (PMC13081499; doi:10.1186/s12944-026-02944-z)
Supplement: Supplementary file 1 — Additional file 1. Supplemental text, figures and tables. [file 12944_2026_2944_MOESM1_ESM.docx]

Supplementary material to

**Serum metabolite patterns of adipose tissue distribution and body composition subphenotypes**

Juliane Maushagen^1^, Elena Grune^1,2^, Christopher L. Schlett^1^, Lena Sophie Kiefer^3,4^, Karsten Suhre^5,6,7^, Jerzy Adamski^8,9,10^, Rui Wang-Sattler^11,12^, Annette Peters^2,11,13,14^, Fabian Bamberg^1^, Susanne Rospleszcz^1,^*

1. Department of Diagnostic and Interventional Radiology, Medical Center - University of Freiburg, Faculty of Medicine, University of Freiburg, Freiburg, Germany.
2. Institute of Epidemiology, Helmholtz Munich, Neuherberg, Germany.
3. Department of Diagnostic and Interventional Radiology, Eberhard Karls University of Tuebingen, Tuebingen, Germany.
4. Department of Nuclear Medicine and Clinical Molecular Imaging, Eberhard Karls University of Tuebingen, Tuebingen, Germany
5. Bioinformatics Core, Weill Cornell Medicine-Qatar, Cornell University, Education City, Doha 24144, Qatar
6. Department of Physiology and Biophysics, Weill Cornell Medical College, New York, NY 10065, USA
7. Caryl and Israel Englander Institute for Precision Medicine, New York, NY 10021, USA
8. Institute of Experimental Genetics, Helmholtz Munich, Neuherberg, Germany.
9. Department of Biochemistry, Yong Loo Lin School of Medicine, National University of Singapore, Singapore, Singapore.
10. Institute of Biochemistry, Faculty of Medicine, University of Ljubljana, Ljubljana, Slovenia.
11. German Center for Diabetes Research (DZD), München-Neuherberg, Germany.
12. Institute of Translational Genomics, Helmholtz Munich, Neuherberg, Germany.
13. Chair of Epidemiology, Institute for Medical Information Processing, Biometry, and Epidemiology (IBE), Medical Faculty, Ludwig-Maximilians-Universität (LMU), München, Germany.
14. German Center for Cardiovascular Disease Research (DZHK), Munich Heart Alliance, Munich, Germany.

**Supplementary text**

**Adipose tissue quantification**

Visceral adipose tissue (VAT) and subcutaneous adipose tissue (SAT) were quantified, in liters [l], from femoral head to diaphragm and to cardiac apex, respectively [1]. Pancreas fat fraction (PF) was quantified in proton density fat fraction [PDFF], is given in percentage and measured as total mean pancreas fat fraction as well as separated into the regions pancreas head (PFcap), body (PFcor) and tail (PFcau) [1]. Bone marrow fat fraction was measured in L1 and L2 vertebrae and quantified as the mean intensity of the fat image divided by the mean of the fat and water image [2]. Adipose tissue in muscles was assessed for the skeletal muscles in the sequence originally used for liver fat quantification and is given as PDFF in percentage [3]. The quantification was conducted on the cellular level and for the whole muscle. Intracellular fat fraction (INTF), the sum of intra- and extracellular fat fraction (TOTF) [4] and the total skeletal muscle fat fraction (SMF) were measured [3], in the right and left rectus abdominis (INTFra , TOTFra, SMFra), psoas major (INTFpm, TOTFpm, SMF pm), quadratus lumborum (INTFql, TOTFql, SMFql) and autochthonous back muscles (INTFab, TOTFab, SMFab). For the heart, adipose tissue were quantified as epicardial (EPCAT) and pericardial (PECAT) fat and measured as area [cm^2^] during maximal systole [5]. Paracardial (PACAT) fat was derived by subtracting the epicardial fat from the pericardial fat [6]. Kidney fat fraction was calculated as fat fraction in the renal sinus (RSF) [%] and the proportion of RSF of the total kidney volume (RF). The kidney parameters are based on the calculations of the entire kidneys, renal cortex, medulla, and pelvis as described in Notohamiprodjo et al. [7].

**Bioelectrical-impedance measurement**

The body impedance analyzer (BIA 2000-S, Data-Input, Pöcking, Germany) with a measurement frequency of 50 kHz at 0.8mA was used to measure resistance and reactance, which were used to calculated the fat free mass according to Kyle (FFM = −4.104 + (0.518 × height^2^/resistance) + (0.231 × weight) + (0.130 × reactance) + (4.229 × sex: men = 1, women = 0) [8] and body fat by subtracting FFM from body weight. Subsequently FMI and LMI were derived by dividing FFM and body fat by the square of body height [9].

**Supplementary Figures**


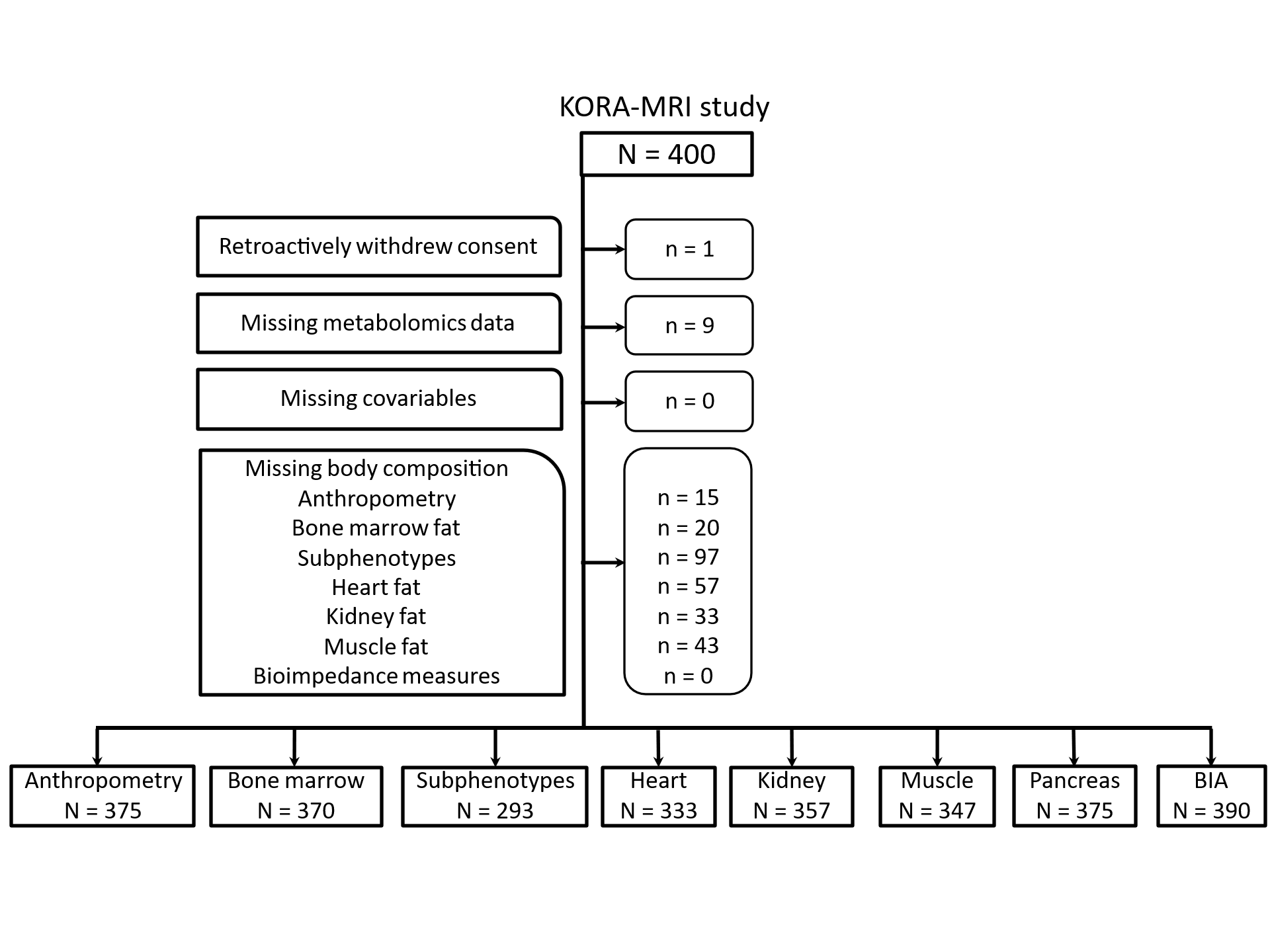


**Supplementary Figure 1** Study sample flowchart. According to missing data in adipose tissue depots, slightly different sample sizes were derived.

**
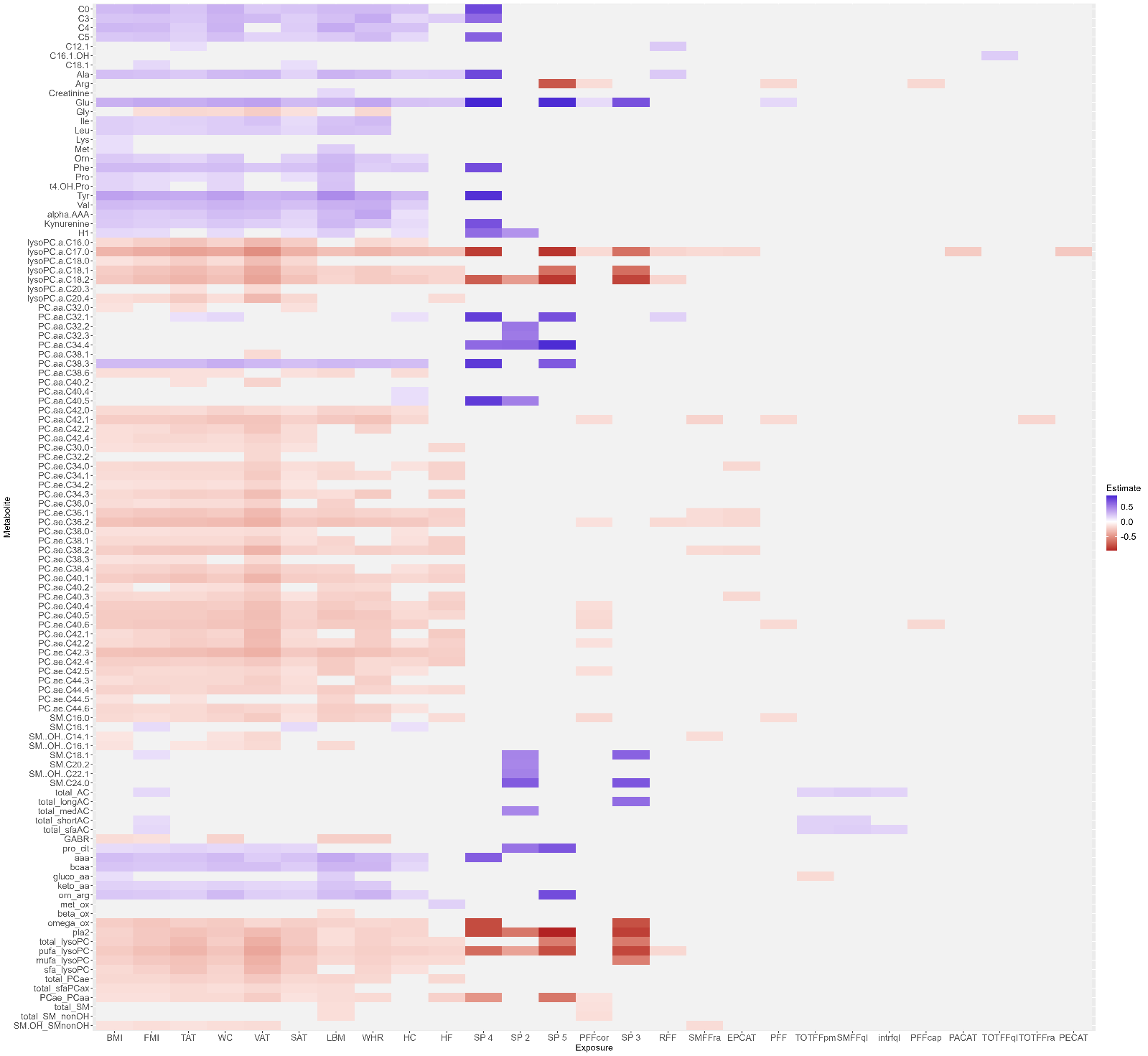
**

**Supplementary Figure 2** Heatmap for associations of metabolites and metabolite indicators with body composition parameters from the fully adjusted model. Significant associations are displayed in colors, with red indicating negative and blue indicating positive associations. Abbreviations: BMI = body mass index, FMI = fat mass index, TAT = total adipose tissue, WC = waist circumference, VAT = visceral adipose tissue, LBM = lean body mass, SAT = subcutaneous fat, WHR = waist to hip ratio, HC = hip circumference, SMRRra = total skeletal muscle fat fraction at rectus abdominis, HF = heart fat, SP4 = subphenotype IV, SP2 = subphenotype II, SP5 = subphenotype V, SP3 = subphenotype III, TOTFFra = the sum of intra- and extracellular fat fraction at rectus abdominis, PFFcor = pancreas corpus fat fraction, RFF = renal fat fraction, TOTFFpm = the sum of intra- and extracellular fat fraction at psoas major, SMFFql = total skeletal muscle fat at quadratus lumborum, intrql = PACAT = Paracardial adipose tissue, TOTFFql = the sum of intra- and extracellular fat fraction at quadratus lumborum, PECAT = pericardial adipose tissue, PFFcap = pancreas caput fat fraction.


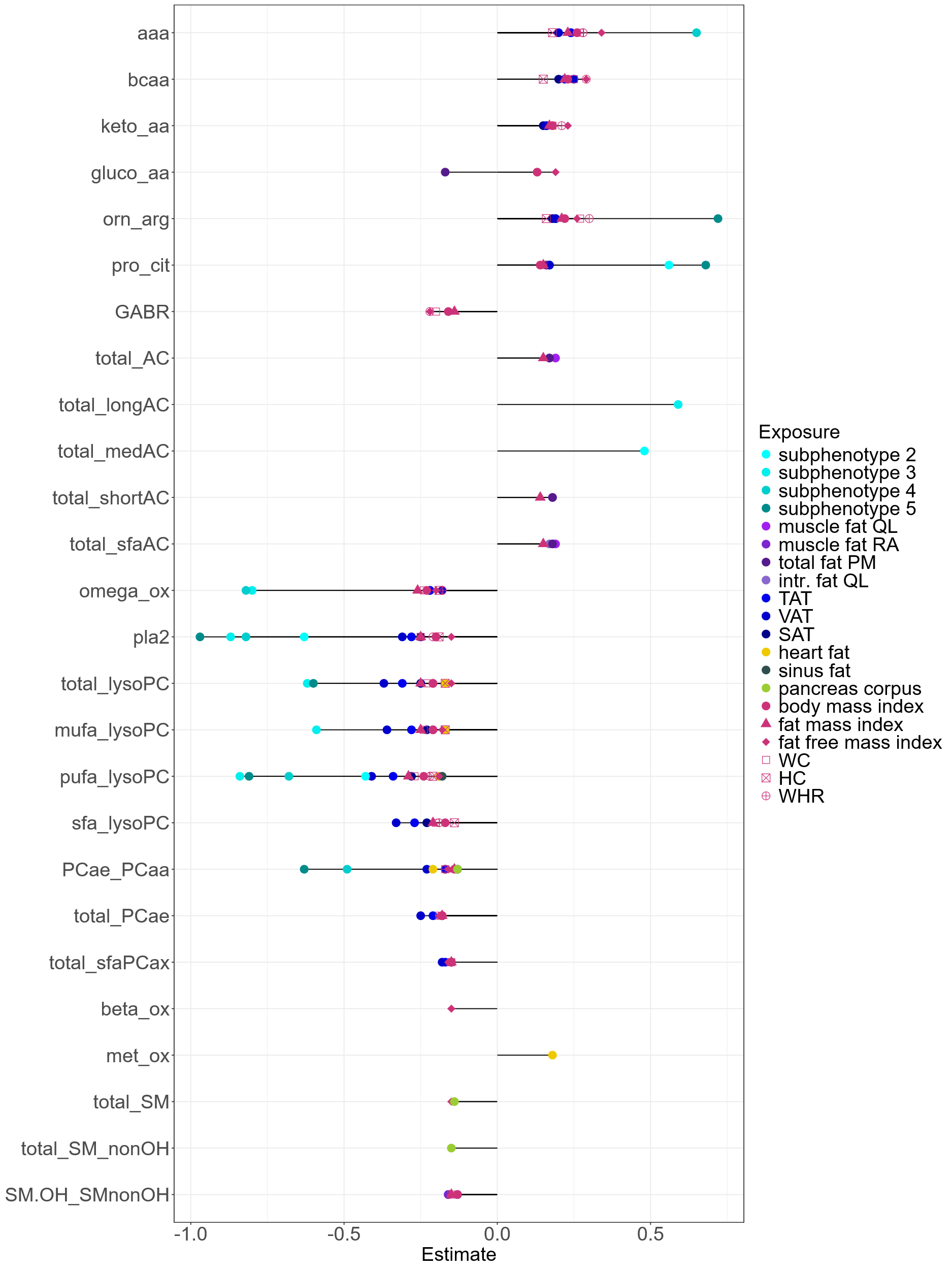


**Supplementary Figure 3** **Metabolite indicators associated with body composition measures.** Lollipop plot showing the significant associations of body composition measures with metabolite indicators of the fully adjusted model (age, sex, body height, smoking, alcohol consumption, physical activity, lipid-lowering medication, diabetes, hypertension and triglycerides). The x-axis shows the effect estimate and the y-axis significant metabolite indicators. Different shades of light blue indicate subphenotypes II-V and purple, green, blue and yellow show MRI-based adipose tissues. Body composition exposures based on BIA- and anthropometric- measures are shown in red by different shapes. Abbreviations: QL = quadratus lumborum; RA = rectus abdominis; TAT = total adipose tissue, VAT = visceral adipose tissue; SAT = subcutaneous adipose tissue; WC = waist circumference; WHR = waist to hip ratio; HC = hip circumference


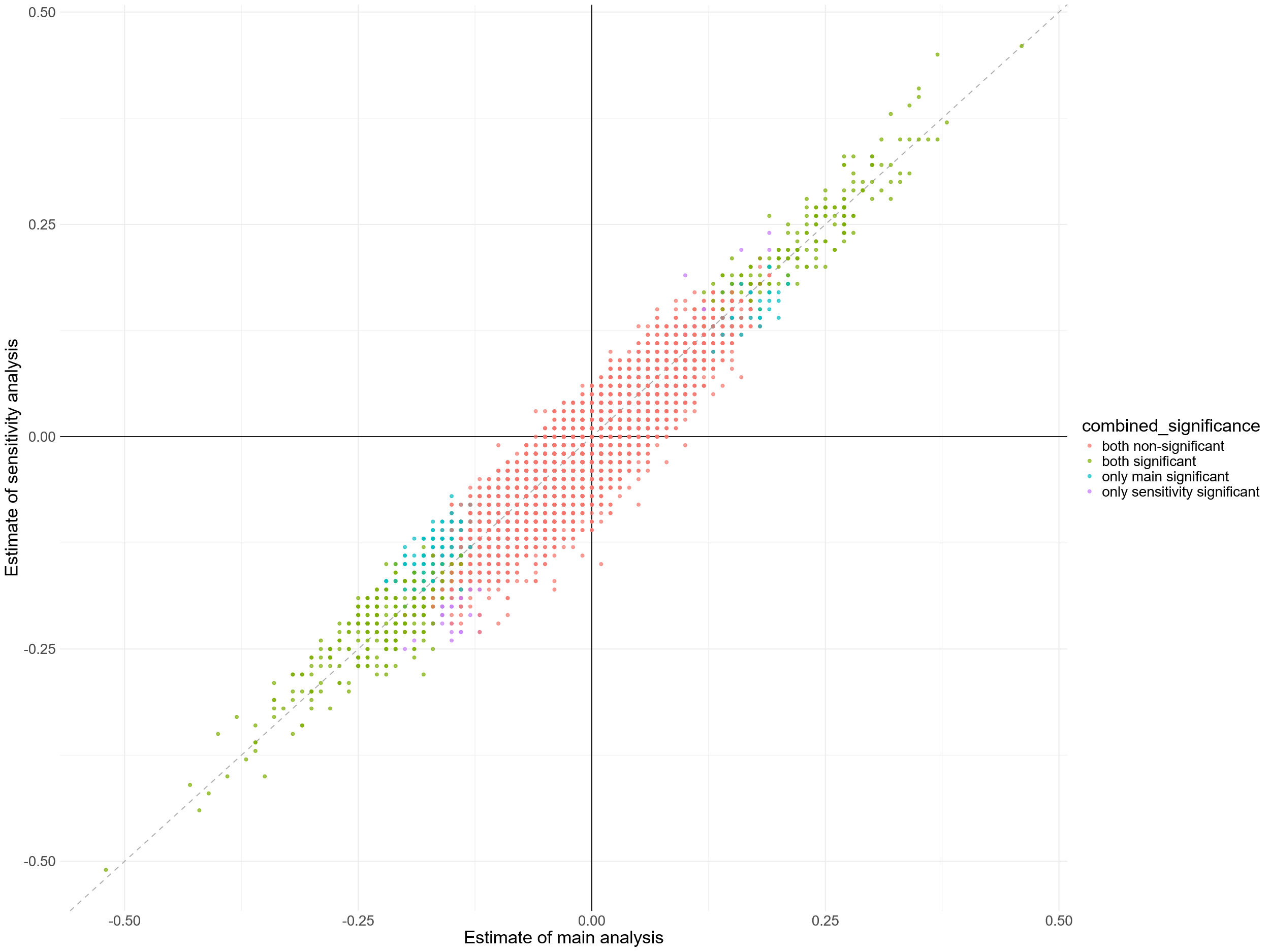


**Supplementary Figure 4** Comparison of effect estimates from the main analysis and from the sensitivity analysis without individuals taking lipid lowering medication. The x-axis shows the effect estimates from the main analysis and the y-axis the effect estimates from the sensitivity analysis. Colors indicate the significance. Effect estimates did not change substantially, and from 6510 associations in total 6263 associations did not change in significance, while for 247 (4%) associations we found a significant FDR-corrected p-value in one of the analyses.


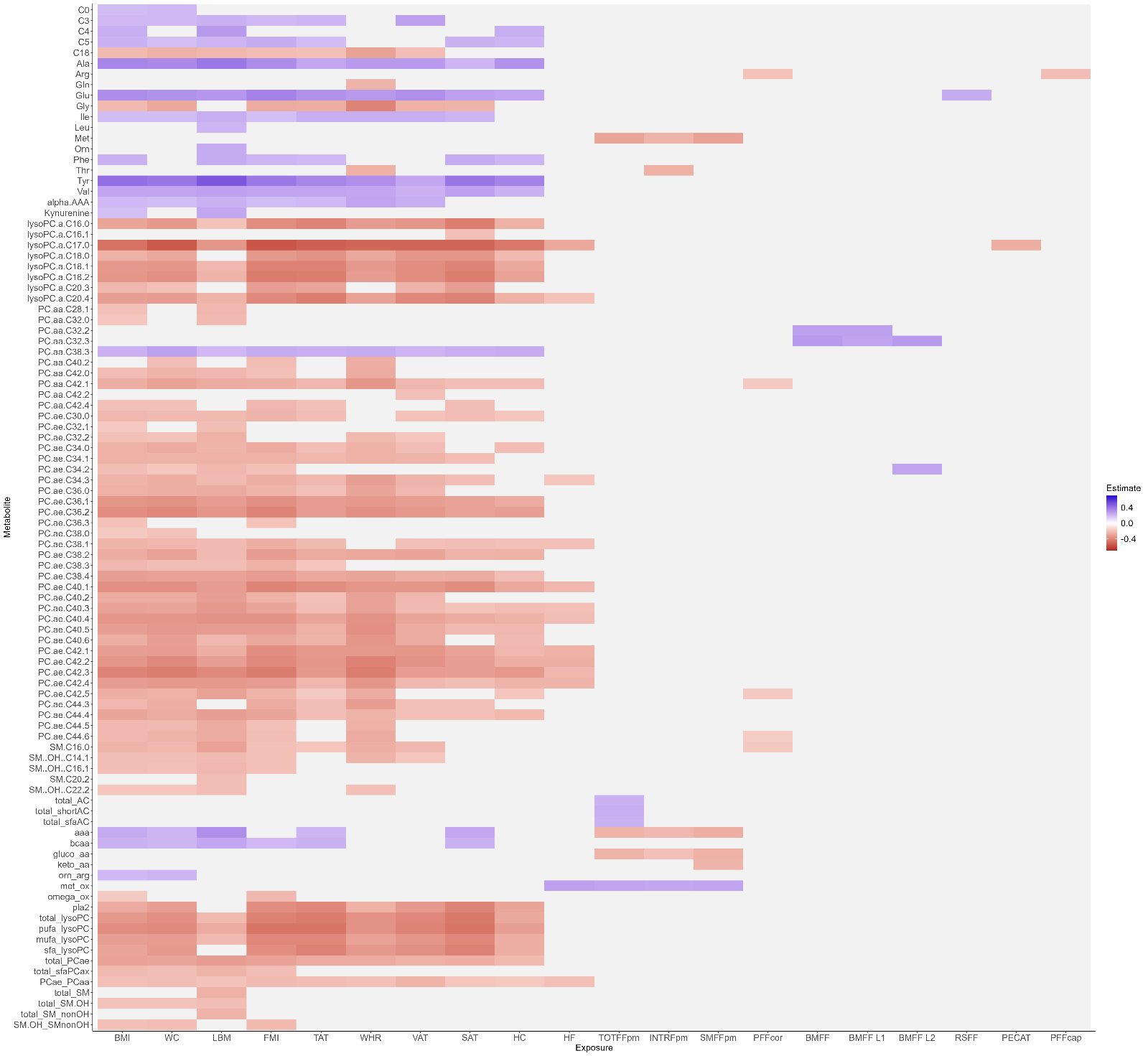


**Supplementary Figure 5** Heatmap for the fully adjusted model in men only. The colors show the effect estimate, where red indicates a negative effect estimate and blue a positive effect estimate. The x-axis shows the body composition exposures that showed any association and the y-axis shows the metabolites that showed any association. Associations were similar to main analyses where men and women were analyzed together.


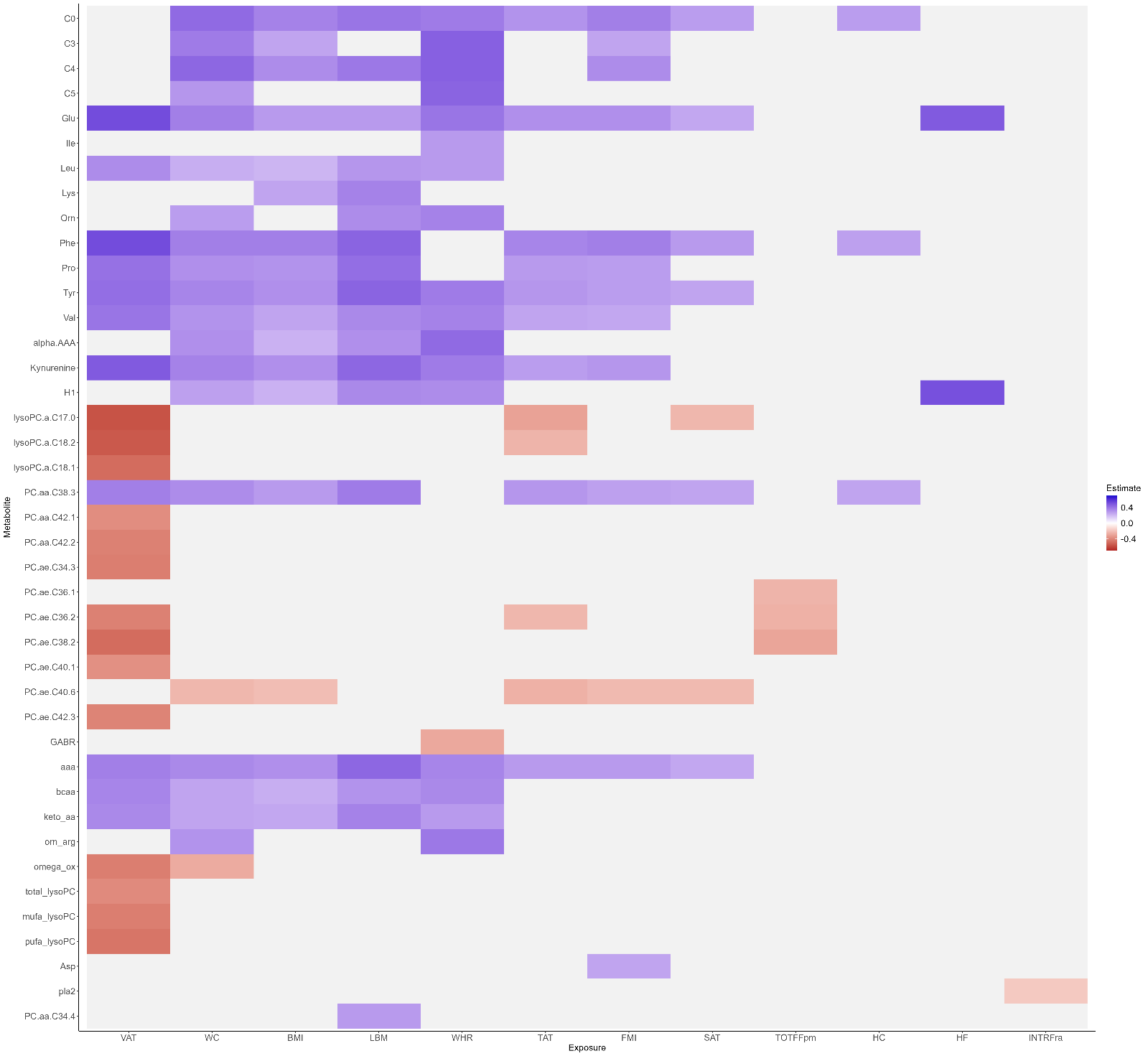


**Supplementary Figure 6** Heatmap for the fully adjusted model in women only. The colors show the effect estimate, where red indicates a negative effect estimate and blue a positive effect estimate. The x-axis shows the body composition exposures that showed any association and the y-axis shows the metabolites that showed any association. In women less metabolites and less exposures were associated with each other. Nominally, associations were similar to the main analysis.


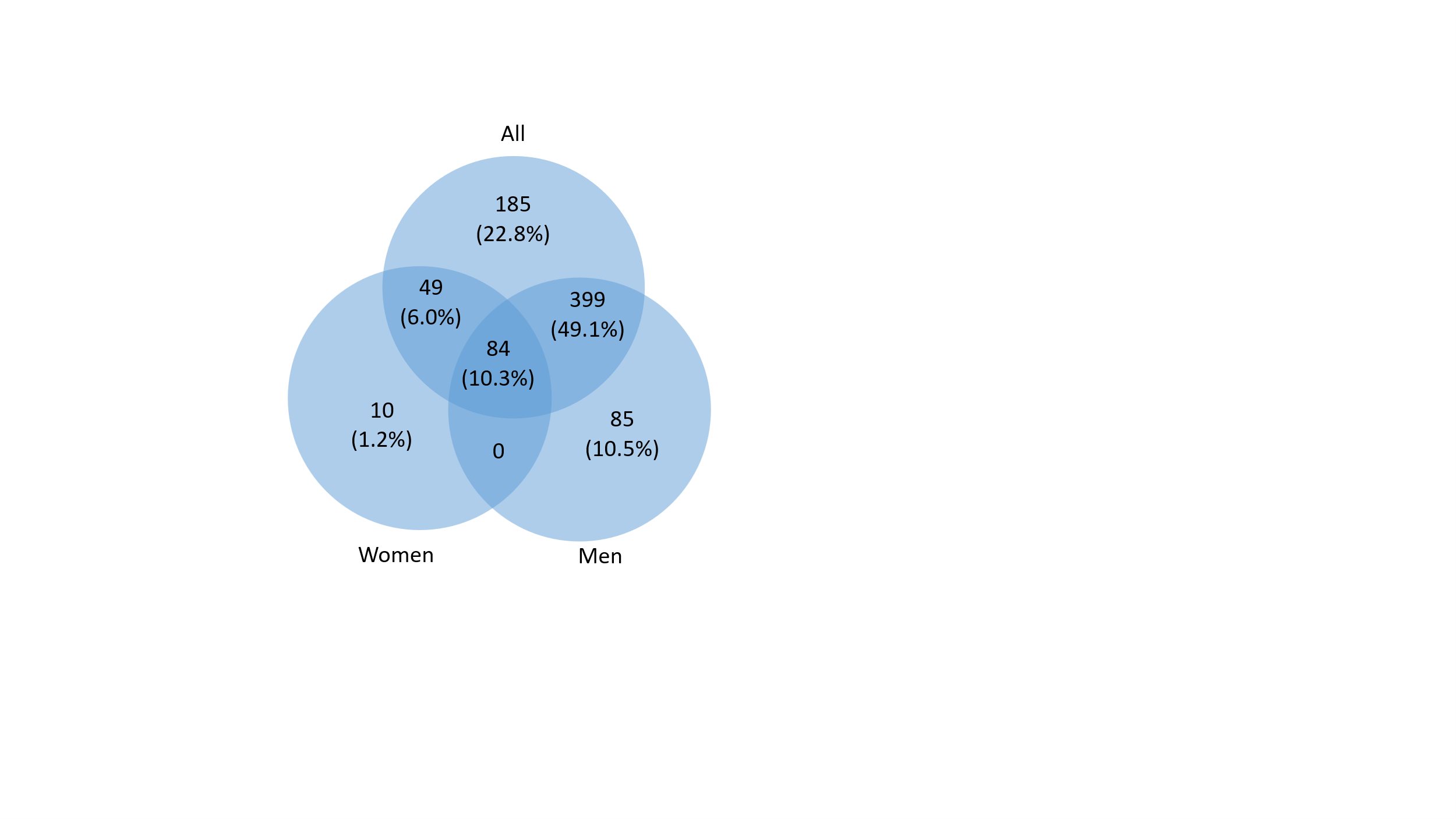


**Supplementary Figure 7** Venn diagram of overlapping significant associations between sex-specific full models and all together. The number of significant metabolite-body composition measure associations are shown. Most associations (49.2%) overlapped between men and all together, while only 1.2% associations were specific to women.


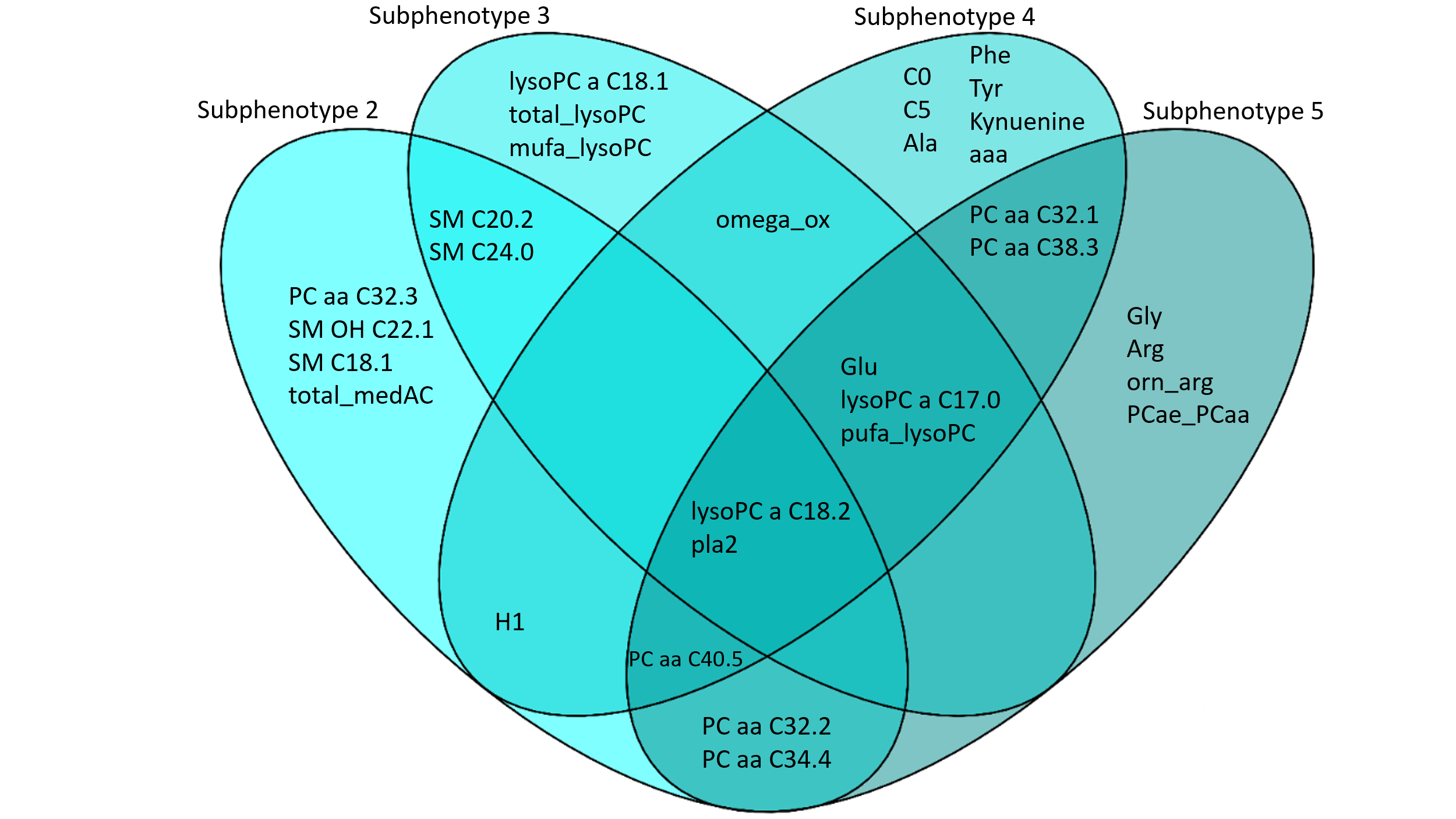


**Supplementary Figure 8** Overlapping metabolites among subphenotype II-V compared to subphenotype I in bootstrapped linear regression models. The model was adjusted for age, sex, body height, diabetes status, hypertension, triglycerides, lipid-lowering medication, smoking, alcohol consumption and physical activity. The color indicates the subphenotype.

Supplementary Tables

**Supplementary Table 1** Description of subphenotypes I – IV

|  | I | II | III | IV | V | p-value |
| --- | --- | --- | --- | --- | --- | --- |
| n | 60 | 106 | 49 | 47 | 31 |  |
| Female sex | 30 (50.0) | 38 (35.8) | 24 (49.0) | 16 (34.0) | 13 (41.9) | 0.249 |
| Age [years] | 48 (7) | 57 (8) | 63 (7) | 55 (7) | 61 (7) | <0.001 |
| BMI [kg/m2] | 24.9 (2.5) | 26.6 (3.7) | 29.5 (3.7) | 32.0 (4.2) | 31.5 (4.5) | <0.001 |
| WHR | 0.9 (0.1) | 0.9 (0.1) | 1.0 (0.1) | 1.0 (0.1) | 1.0 (0.1) | <0.001 |
| Hip circumference [cm] | 102.4 (5.4) | 103.6 (6.8) | 109.4 (6.9) | 112.9 (8.2) | 111.3 (9.8) | <0.001 |
| Waist circumference [cm] | 87.0 (8.8) | 94.9 (11.4) | 103.8 (10.2) | 110.4 (9.6) | 107.6 (10. 9) | <0.001 |
| Physically inactive | 13 (21.7) | 45 (42.5) | 26 (53.1) | 22 (46.8) | 13 (41.9) | 0.011 |
| Alcohol consumption* [g/day] | 7.7 (20.9) | 8.6 (27.1) | 14.3 (25.3) | 5.7 (25.6) | 12.3 (27.1) | 0.65 |
| Smoking behavior |  |  |  |  |  | 0.018 |
| Never smoker | 28 (46.7) | 40 (37.7) | 20 (40.8) | 14 (29.8) | 7 (22.6) |  |
| Former smoker | 20 (33.3) | 37 (34.9) | 22 (44.9) | 27 (57.4) | 20 (64.5) |  |
| Current smoker | 12 (20.0) | 29 (27.4) | 7 (14.3) | 6 (12.8) | 4 (13.0) |  |
| LDL [mg/dl] | 127. 8 (30.1) | 147.8 (32.5) | 142.1 (31.9) | 144.3 (36.3) | 138.3 (30.0) | 0.004 |
| Triglycerides [mg/dl] | 89.0 (43.0) | 128.3 (86.5) | 140.8 (94.9) | 183.7 (109.8) | 154.8 (82.4) | <0.001 |
| Statin intake | 3 (5.0) | 10 (9.4) | 8 (16.3) | 5 (10.6) | 6 (9.4) | 0.189 |
| Hypertension | 5 (8.3) | 21 (19.8) | 25 (51.0) | 18 (38.3) | 18 (58.1) | <0.001 |
| Systolic blood pressure [mmHg] | 112.9 (14.9) | 119.2 (15.2) | 126.3 (13.8) | 129.1 (18.6) | 123.9 (12.9) | <0.001 |
| Diastolic blood pressure [mmHg] | 72.0 (9.3) | 74.3 (9.0) | 75.8 (9.7) | 81.8 (9.2) | 76.9 (8.3) | <0.001 |
| Glycemic status |  |  |  |  |  | <0.001 |
| Normal | 53 (88.3) | 80 (75.5) | 19 (38.8) | 17 (36.2) | 11 (35.5) |  |
| Prediabetes | 6 (10.0) | 17 (16.0) | 20 (40.8) | 20 (42.6) | 10 (32.3) |  |
| Diabetes | 1 (1.7) | 9 (8.5) | 10 (20.4) | 10 (21.3) | 10 (32.3) |  |
| Antidiabetic medication | none | 5 (4.7) | 6 (12.2) | 4 (8.5) | 8 (15.8) | <0.001 |
| hsCRP* [mg/l] | 0.65 (0.57) | 1.12 (1.3) | 1.6 (2.0) | 1.91 (2.5) | 1. 7 (4.4) | <0.001 |
| GFR [ml/min/1.73 m2] | 98.1 (11.2) | 91.3 (13.8) | 85.8 (14.0) | 86.1 (12.8) | 82.7 (13.3) | <0.001 |
| **Body composition** | | | | | | |
| **Body fat** | | | | | | |
| TAT [l] | 7.4 (1.9) | 10.9 (3.2) | 15. 7 (4.0) | 17.8 (4.2) | 17.3 (4.4) | <0.001 |
| VAT [l] | 1.9 (1.1) | 4.0 (1.8) | 6.0 (2.5) | 6.7 (2.1) | 7.2 (2.5) | <0.001 |
| SAT [l] | 5.5 (1.6) | 6.9 (2.2) | 9.6 (3.1) | 11.2 (4.0) | 10.1 (3.8) | <0.001 |
| **Bone marrow fat** | | | | | | |
| BMF [%] | 0.4 (0.1) | 0.6 (0.1) | 0.6 (0.1) | 0.5 (0.1) | 0.6 (0.1) | <0.001 |
| BMF L1 [%] | 0.4 (0.1) | 0.6 (0.1) | 0.6 (0.1) | 0.5 (0.1) | 0.6 (0.1) | <0.001 |
| BMF L2 [%] | 0.4 (0.1) | 0.6 (0.1) | 0.6 (0.1) | 0.5 (0.1) | 0.6 (0.1) | <0.001 |
| **Cardiac fat** | | | | | | |
| HF [ml] | 61.5 (32.9) | 116.6 (52.3) | 165.9 (73.7) | 170.3 (69.5) | 191.3 (75.2) | <0.001 |
| EPCAT [cm2] | 5.9 (3.1) | 8.4 (3.4) | 10.8 (4.1) | 10.9 (5.2) | 12.0 (6.8) | <0.001 |
| PECAT [cm2] | 16.7 (9.3) | 27.3 (11.5) | 37.3 (16.2) | 38.6 (18.0) | 42.1 (22.3) | <0.001 |
| PACAT [cm2] | 10.9 (7.2) | 18.9 (9.5) | 26.5 (14.2) | 27. 8 (15.4) | 30.1 (17.3) | <0.001 |
| **Pancreatic fat** | | | | | | |
| PF [%] | 3.9 (3.3) | 5.9 (3.8) | 8.3 (4.2) | 6.8 (3.4) | 24.6 (8.6) | <0.001 |
| PFcap [%] | 3. 7 (3.0) | 6.0 (4.1) | 7.8 (4.5) | 7.2 (3.7) | 25.0 (9.8) | <0.001 |
| PFcor [%] | 4.0 (3.8) | 6.1 (3.9) | 8.4 (4.7) | 6.8 (3.8) | 24.7 (9.0) | <0.001 |
| PFcau [%] | 4.0 (3.7) | 5.7 (4.0) | 8.6 (4.9) | 6.6 (3.7) | 24.0 (8.2) | <0.001 |
| **Renal fat** | | | | | | |
| RF [%] | 0.1 (0.0) | 0.1 (0.0) | 0.1 (0.0) | 0.1 (0.0) | 0.1 (0.0) | <0.001 |
| RSF [%] | 0.6 (0.1) | 0.6 (0.1) | 0.7 (0.1) | 0.7 (0.1) | 0.7 (0.1) | <0.001 |
| **Muscle fat** | | | | | | |
| SMF pm [%] | 5.0 (1.4) | 7.1 (2.3) | 11.3 (4.2) | 7.2 (2.3) | 8.4 (3.5) | <0.001 |
| SMF ql [%] | 4.5 (1.8) | 6.4 (2.7) | 11.8 (4.5) | 5.0 (2.0) | 7.3 (4.2) | <0.001 |
| SMF ar [%] | 11.4 (4.4) | 16.8 (6.0) | 26.3 (7.2) | 15.4 (5.6) | 20.9 (8.7) | <0.001 |
| SMF ra [%] | 11.2 (7.7) | 13.1 (7.7) | 21.5 (12.3) | 14.9 (9.7) | 19.3 (10.4) | <0.001 |
| SMF weighted per area [%] | 9.4 (3.1) | 13.3 (4.1) | 21.4 (5.1) | 12.7 (4.2) | 16.9 (6.0) | <0.001 |
| TOTF pm [%] | 5.0 (1.4) | 7.2 (2.1) | 11.3 (3.8) | 7.6 (2.4) | 8.8 (3.4) | <0.001 |
| TOTF ql [%] | 4.5 (1.7) | 6.6 (3.6) | 10.6 (6.1) | 5.8 (2.4) | 8.1 (5.4) | <0.001 |
| TOTF ar [%] | 10.5 (4.1) | 15.7 (6.0) | 25.4 (8.6) | 14.8 (5.8) | 20.3 (9.8) | <0.001 |
| TOTF ra [%] | 11.4 (7.4) | 13.5 (7.5) | 22.9 (15.5) | 17.1 (8.6) | 19.9 (9.6) | <0.001 |
| INTRF pm [%] | 3.9 (0.9) | 5.4 (1.6) | 7.6 (2.3) | 5.5 (1.7) | 6.4 (2.1) | <0.001 |
| INTRF ql [%] | 3.2 (1.1) | 4.3 (1.5) | 5.9 (1.9) | 3.7 (1.3) | 4.5 (1.7) | <0.001 |
| INTRF ar [%] | 5.7 (1.4) | 7.7 (2.1) | 11.0 (3.0) | 7.5 (2.5) | 9.4 (3.4) | <0.001 |
| INTRF ra [%] | 6.0 (2.6) | 7.0 (2.8) | 10.3 (4.5) | 7.9 (3.3) | 8.7 (2.2) | <0.001 |
| **Bioelectrical-impedance measurement** | | | | | | |
| Fat mass index [kg/m2] | 7.1 (1.8) | 8.1 (2.2) | 10.6 (2.6) | 11.7 (3.3) | 11.6 (3.3) | <0.001 |
| Lean body mass index [kg/m2] | 17.8 (2.1) | 18.5 (2.4) | 18.9 (2.4) | 20.4 (2.1) | 19.9 (2.2) | <0.001 |

Abbreviations: BMI = body mass index, WHR = waist to hip ratio, LDL = low density lipoprotein, hsCRP = high sensitive C-reactive protein, GFR = glomerulus filtration rate, TAT = total adipose tissue; VAT = visceral adipose tissue; SAT = subcutaneous adipose tissue; BMF L1/L2 = bone marrow fat at L1/L2; HF = heart fat; EPCAT = epicardial fat; PECAT = pericardial fat; PACAT = Paracardial fat; SMF = skeletal muscle fat; TOTF = intra- and extracellular fat; INTRF = intracellular fat; pm = psoas major; ql = quadratus lumborum; ar = autochthonous back muscles; ra = rectus abdominis; RF = fenal fat; RSF = renal sinus fat; PF = mean pancreatic fat; PFcap = pancreatic fat at capus; PFcor = pancreatic fat at corpus; PFcau = pancreatic fat at cauda

**Supplementary Table 2** Metabolite groups, names and abbreviations adapted from Maushagen et al. [10].

| Metabolite group | Abbreviation | Biochemical name | HMDB IDs |
| --- | --- | --- | --- |
| Carnitines | C0 | Carnitine | HMDB0000062 |
|  | C2 | Acetylcarnitine | HMDB0000201 |
|  | C3 | Propionylcarnitine | HMDB0000824 |
|  | C3-DC C4-OH | Hydroxypropionylcarnitine | HMDB0002095  HMDB0013127 |
|  | C4 | Butyrylcarnitine | HMDB0002013  HMDB0000736 |
|  | C4:1 | Butenylcarnitine | HMDB0013126 |
|  | C5 | Valerylcarnitine | HMDB0000378  HMDB0000688  HMDB0013128 |
|  | C7-DC | Pimelylcarnitine |  |
|  | C8 | Octanoylcarnitine | HMDB0000791 |
|  | C9 | Nonaylcarnitine | HMDB0006320 |
|  | C10 | Decanoylcarnitine | HMDB0000651 |
|  | C10:1 | Decenoylcarnitine |  |
|  | C12 | Dodecanoylcarnitine | HMDB0002250 |
|  | C12:1 | Dodecenoylcarnitine |  |
|  | C14 | Tetradecanoylcarnitine |  |
|  | C14:1 | Tetradecenoylcarnitine | HMDB0002014 |
|  | C14:1-OH | Hydroxytetradecenoylcarnitine |  |
|  | C14:2 | Tetradecadienylcarnitine |  |
|  | C14:2-OH | Hydroxytetradecadienylcarnitine |  |
|  | C16 | Hexadecanylcarnitine | HMDB0000222 |
|  | C16:1-OH | Hydroxyhexadecenoylcarnitine |  |
|  | C16:2 | Hexadecadienylcarnitine |  |
|  | C18 | Octadecanoylcarnitine | HMDB0000848 |
|  | C18:1 | Octadecenoylcarnitine | HMDB0006464 |
|  | C18:2 | Octadecadienylcarnitine | HMDB0006461 |
| Amino acids | Ala | Alanine | HMDB0001310  HMDB0000161  HMDB0000056 |
|  | Arg | Arginine | HMDB0003416  HMDB0000517 |
|  | Asn | Asparagine | HMDB0033780  HMDB0000168 |
|  | Asp | Aspartate | HMDB0006483  HMDB0000191 |
|  | Cit | Citrulline | HMDB0000904 |
|  | Gln | Glutamine | HMDB0003423  HMDB0000641 |
|  | Glu | Glutamate | HMDB0003339  HMDB0000148 |
|  | Gly | Glycine | HMDB0000123 |
|  | His | Histidine | HMDB0000177 |
|  | Ile | Isoleucine | HMDB0000172 |
|  | Leu | Leucine | HMDB0000687  HMDB0003640  HMDB0003640 |
|  | Lys | Lysine | HMDB0003405  HMDB0000182 |
|  | Met | Methionine | HMDB0000696 |
|  | Orn | Ornithine | HMDB0003374  HMDB0000214 |
|  | Phe | Phenylalanine | HMDB0000159 |
|  | Pro | Proline | HMDB0003411  HMDB0000162 |
|  | Ser | Serine | HMDB0003406  HMDB0000187 |
|  | Thr | Threonine | HMDB0004041  HMDB0000167 |
|  | Trp | Tryptophan | HMDB0013609  HMDB0000929 |
|  | Tyr | Tryosine | HMDB0000158 |
|  | Val | Valine | HMDB0000883 |
| Biogenic amines | Ac.Orn | Acetylornithine | HMDB0003357 |
|  | ADMA | Asymmetric dimethylarginine | HMDB0001539 |
|  | alpha.AAA | alpha-Aminoadipic acid | HMDB0000510 |
|  | Creatinine | Creatinine | HMDB0000562 |
|  | Kynurenine | Kynurenine | HMDB0000684 |
|  | Met.SO | Methioninesulfoxide | HMDB0002005 |
|  | SDMA | Symmetric dimethylarginine | HMDB0003334 |
|  | Spermidine | Spermidine | HMDB0001257 |
|  | t4.OH.Pro | 4-Hydroxyproline | HMDB0000725 |
|  | Taurine | Taurine | HMDB0000251 |
|  | total.DMA | Total dimethylarginine |  |
| Lysophosphatidylcholines | lysoPC a C16:0 | lysoPhosphatidylcholine acyl C16:0 | HMDB0010382 |
|  | lysoPC a C16:1 | lysoPhosphatidylcholine acyl C16:1 | HMDB0010383 |
|  | lysoPC a C17:0 | lysoPhosphatidylcholine acyl C17:0 | HMDB0012108 |
|  | lysoPC a C18:0 | lysoPhosphatidylcholine acyl C18:0 | HMDB0011128 |
|  | lysoPC a C18:1 | lysoPhosphatidylcholine acyl C18:1 | HMDB0002815 |
|  | lysoPC a C18:2 | lysoPhosphatidylcholine acyl C18:2 | HMDB0010386 |
|  | lysoPC a C20:3 | lysoPhosphatidylcholine acyl C20:3 | HMDB0010393  HMDB0010394 |
|  | lysoPC a C20:4 | lysoPhosphatidylcholine acyl C20:4 | HMDB0010395  HMDB0010396 |
| Diacylphosphatidylcholines | PC aa C28:1 | Phosphatidylcholine diacyl C28:1 |  |
|  | PC aa C30:0 | Phosphatidylcholine diacyl C30:0 | HMDB0007965  HMDB0007934 |
|  | PC aa C32:0 | Phosphatidylcholine diacyl C30:0 | HMDB0007871  HMDB0008031  HMDB0000564 |
|  | PC aa C32:1 | Phosphatidylcholine diacyl C32:1 | HMDB0007872  HMDB0008097  HMDB0007969  HMDB0007873 |
|  | PC aa C32:2 | Phosphatidylcholine diacyl C32:2 | HMDB0008002  HMDB0007874 |
|  | PC aa C32:3 | Phosphatidylcholine diacyl C32:3 | HMDB0007876 |
|  | PC aa C34:1 | Phosphatidylcholine diacyl C34:1 | HMDB0008035  HMDB0008003  HMDB0007971  HMDB0007972 |
|  | PC aa C34:2 | Phosphatidylcholine diacyl C34:2 | HMDB0008133  HMDB0008101  HMDB0008004  HMDB0008005  HMDB0007973 |
|  | PC aa C34:3 | Phosphatidylcholine diacyl C34:3 | HMDB0008006  HMDB0007974  HMDB0007975 |
|  | PC aa C34:4 | Phosphatidylcholine diacyl C34:4 | HMDB0007976  HMDB0007883 |
|  | PC aa C36:1 | Phosphatidylcholine diacyl C36:1 | HMDB0008102  HMDB0008037  HMDB0008069  HMDB0008038 |
|  | PC aa C36:2 | Phosphatidylcholine diacyl C36:2 | HMDB0008135  HMDB0008070  HMDB0000593  HMDB0008039 |
|  | PC aa C36:3 | Phosphatidylcholine diacyl C36:3 | HMDB0008040  HMDB0007980  HMDB0007981  HMDB0008105 |
|  | PC aa C36:4 | Phosphatidylcholine diacyl C36:4 | HMDB0008429  HMDB0008170  HMDB0008203  HMDB0008138  HMDB0008106  HMDB0008107  HMDB0008042  HMDB0007982 |
|  | PC aa C36:5 | Phosphatidylcholine diacyl C36:5 | HMDB0008015  HMDB0007984 |
|  | PC aa C36:6 | Phosphatidylcholine diacyl C36:3 | HMDB0008206  HMDB0007892 |
|  | PC aa C38:0 | Phosphatidylcholine diacyl C38:0 | HMDB0008755  HMDB0008528  HMDB0008267  HMDB0008043  HMDB0007985  HMDB0007893 |
|  | PC aa C38:1 | Phosphatidylcholine diacyl C38:1 | HMDB0008268  HMDB0008269  HMDB0008109  HMDB0008044  HMDB0007986  HMDB0007894 |
|  | PC aa C38:3 | Phosphatidylcholine diacyl C38:3 | HMDB0008046  HMDB0008047 |
|  | PC aa C38:4 | Phosphatidylcholine diacyl C38:4 | HMDB0008112  HMDB0008048  HMDB0007988 |
|  | PC aa C38:5 | Phosphatidylcholine diacyl C38:5 | HMDB0008114  HMDB0008050  HMDB0007989  HMDB0007990 |
|  | PC aa C38:6 | Phosphatidylcholine diacyl C38:6 | HMDB0008725  HMDB0008499  HMDB0008434  HMDB0008147  HMDB0008083  HMDB0008116  HMDB0007991 |
|  | PC aa C40:2 | Phosphatidylcholine diacyl C40:2 | HMDB0008308  HMDB0008276 |
|  | PC aa C40:3 | Phosphatidylcholine diacyl C40:3 |  |
|  | PC aa C40:4 | Phosphatidylcholine diacyl C40:4 | HMDB0008628  HMDB0008279  HMDB0008054 |
|  | PC aa C40:5 | Phosphatidylcholine diacyl C40:5 | HMDB0008120  HMDB0008055  HMDB0008056 |
|  | PC aa C40:6 | Phosphatidylcholine diacyl C40:6 | HMDB0008089  HMDB0008122  HMDB0008057 |
|  | PC aa C42:0 | Phosphatidylcholine diacyl C42:0 | HMDB0008760  HMDB0008537  HMDB0008282  HMDB0008058 |
|  | PC aa C42:1 | Phosphatidylcholine diacyl C42:1 | HMDB0008762  HMDB0008538  HMDB0008283  HMDB0008124  HMDB0008059 |
|  | PC aa C42:2 | Phosphatidylcholine diacyl C42:2 |  |
|  | PC aa C42:4 | Phosphatidylcholine diacyl C42:4 |  |
|  | PC aa C42:5 | Phosphatidylcholine diacyl C42:5 | HMDB0008287 |
|  | PC aa C42:6 | Phosphatidylcholine diacyl C42:6 | HMDB0008288 |
| Acylalkylphosphatidylcholines | PC ae C30:0 | Phosphatidylcholine acyl-alkyl C30:0 | HMDB0013341 |
|  | PC ae C32:1 | Phosphatidylcholine acyl-alkyl C32:1 |  |
|  | PC ae C32:2 | Phosphatidylcholine acyl-alkyl C32:2 |  |
|  | PC ae C34:0 | Phosphatidylcholine acyl-alkyl C34:0 | HMDB0013405 |
|  | PC ae C34:1 | Phosphatidylcholine acyl-alkyl C34:1 |  |
|  | PC ae C34:2 | Phosphatidylcholine acyl-alkyl C34:2 | HMDB0011151 |
|  | PC ae C34:3 | Phosphatidylcholine acyl-alkyl C34:3 | HMDB0011211 |
|  | PC ae C36:0 | Phosphatidylcholine acyl-alkyl C36:0 | HMDB0013417  HMDB0013406 |
|  | PC ae C36:1 | Phosphatidylcholine acyl-alkyl C36:1 |  |
|  | PC ae C36:2 | Phosphatidylcholine acyl-alkyl C36:2 | HMDB0011243 |
|  | PC ae C36:3 | Phosphatidylcholine acyl-alkyl C36:3 |  |
|  | PC ae C36:4 | Phosphatidylcholine acyl-alkyl C36:4 |  |
|  | PC ae C36:5 | Phosphatidylcholine acyl-alkyl C36:5 | HMDB0011220 |
|  | PC ae C38:0 | Phosphatidylcholine acyl-alkyl C38:0 | HMDB0013419  HMDB0013408 |
|  | PC ae C38:1 | Phosphatidylcholine acyl-alkyl C38:1 |  |
|  | PC ae C38:2 | Phosphatidylcholine acyl-alkyl C38:2 |  |
|  | PC ae C38:3 | Phosphatidylcholine acyl-alkyl C38:3 |  |
|  | PC ae C38:4 | Phosphatidylcholine acyl-alkyl C38:4 | HMDB0013420 |
|  | PC ae C38:5 | Phosphatidylcholine acyl-alkyl C38:5 | HMDB0013432 |
|  | PC ae C38:6 | Phosphatidylcholine acyl-alkyl C38:6 | HMDB0013409 |
|  | PC ae C40:1 | Phosphatidylcholine acyl-alkyl C40:1 |  |
|  | PC ae C40:2 | Phosphatidylcholine acyl-alkyl C40:2 |  |
|  | PC ae C40:3 | Phosphatidylcholine acyl-alkyl C40:3 |  |
|  | PC ae C40:4 | Phosphatidylcholine acyl-alkyl C40:4 |  |
|  | PC ae C40:5 | Phosphatidylcholine acyl-alkyl C40:5 |  |
|  | PC ae C40:6 | Phosphatidylcholine acyl-alkyl C40:6 | HMDB0013421 |
|  | PC ae C42:1 | Phosphatidylcholine acyl-alkyl C42:1 |  |
|  | PC ae C42:2 | Phosphatidylcholine acyl-alkyl C42:2 |  |
|  | PC ae C42:3 | Phosphatidylcholine acyl-alkyl C42:3 |  |
|  | PC ae C42:4 | Phosphatidylcholine acyl-alkyl C42:4 |  |
|  | PC ae C42:5 | Phosphatidylcholine acyl-alkyl C42:5 |  |
|  | PC ae C44:3 | Phosphatidylcholine acyl-alkyl C44:3 |  |
|  | PC ae C44:4 | Phosphatidylcholine acyl-alkyl C44:4 |  |
|  | PC ae C44:5 | Phosphatidylcholine acyl-alkyl C44:5 |  |
|  | PC ae C44:6 | Phosphatidylcholine acyl-alkyl C44:6 |  |
| Sphingomyelins | SM (OH) C14:1 | Hydroxysphingomyeline C14:1 |  |
|  | SM (OH) C16:1 | Hydroxysphingomyeline C16:1 |  |
|  | SM (OH) C22:1 | Hydroxysphingomyeline C22:1 |  |
|  | SM (OH) C22:2 | Hydroxysphingomyeline C22:2 |  |
|  | SM (OH) C24:1 | Hydroxysphingomyeline C24:1 |  |
|  | SM C16:0 | Sphingomyeline C16:0 |  |
|  | SM C16:1 | Sphingomyeline C16:1 |  |
|  | SM C18:0 | Sphingomyeline C18:0 |  |
|  | SM C18:1 | Sphingomyeline C18:1 | HMDB0012100  HMDB0012101 |
|  | SM C20:2 | Sphingomyeline C20:2 |  |
|  | SM C24:0 | Sphingomyeline C24:0 |  |
|  | SM C24:1 | Sphingomyeline C24:1 | HMDB0012107 |
| Hexoses | H1 | Hexoses | HMDB0000143  HMDB0003345  HMDB0003449  HMDB0000516  HMDB0000660  HMDB0000122  HMDB0000169  HMDB0003418  HMDB0012326 |

**Supplementary Table 3** Metabolite indicators including, name, short name and the formula adapted from Maushagen et al. [10].

| **Metabolite indicator** | **Short name** | **Formula** |
| --- | --- | --- |
| Fischer Ratio | fischer_ratio | (Ile + Leu + Val) / (Phe + Trp + Tyr) |
| Global Arginine Bioavailability Ratio | GABR | Arg/ (Orn + Cit) |
| Ratio of Proline to Citrulline | pro_cit | Pro / Cit |
| Sum of Aromatic Amino Acids | aaa | Phe + Trp + Tyr |
| Sum of Branched-Chain Amino Acids | bcaa | Ile + Leu + Val |
| Sum of Solely Glucogenic Amino Acids | gluco_aa | Ala + Arg + Asn + Asp + Gln + Glu + Gly + His + Met + Pro + Ser + Thr + Val |
| Sum of Solely Ketogenic Amino Acids | keto_aa | Leu + Lys |
| Asymmetrical Arginine Methylation | adma_arg | ADMA / Arg |
| Methionine Oxidation | met_ox | Met.SO / Met |
| Nitric Oxide-Synthase Activity | cit_arg | Cit / Arg |
| Ornithine Synthesis | orn_arg | Orn / Arg |
| Beta-Oxidation | beta_ox | (C2 + C3) / C0 |
| Sum of Acylcarnitines | total_AC | C2 + C3 + C3.DC..C4.OH. + C4 + C4.1 + C5 + C7.DC + C8 + C9 + C10 + C10.1 + C12 + C12.1 + C14 + C14.1 + C14.1.OH + C14.2 + C14.2.OH + C16 + C16.1.OH + C16.2 + C18 + C18.1 + C18.2 |
| Sum of Long-Chain Acylcarnitines | total_longAC | C14 + C14.1 + C14.1.OH + C14.2 + C14.2.OH + C16 + C16.1.OH + C16.2 + C18 + C18.1 + C18.2 |
| Sum of Medium-Chain Acylcarnitines | total_medAC | C7.DC + C8 + C9 + C10 + C10.1 + C12 + C12.1 |
| Sum of Monounsaturated Fatty Acid Acylcarnitines | total_mufaAC | C4.1 + C10.1 + C12.1 + C14.1 + C14.1.OH + C16.1.OH + C18.1 |
| Sum of Polyunsaturated Fatty Acid Acylcarnitines | total_pufaAC | C14.2 + C14.2.OH + C16.2 + C18.2 |
| Sum of Saturated Fatty Acid Acylcarnitines | total_sfaAC | C2 + C3 + C3.DC..C4.OH. + C4 + C5 + C7.DC + C8 + C9 + C10 + C12 + C14 + C16 + C18 |
| Sum of Short-Chain Acylcarnitines | total_shortAC | C2 + C3 + C3.DC..C4.OH. + C4 + C4.1 + C5 |
| Omega-Oxidation | omega_ox | (C3.DC..C4.OH. + C7.DC) / (C2 + C3 + C3.DC..C4.OH. + C4 + C4.1 + C5 + C7.DC + C8 + C9 + C10 + C10.1 + C12 + C12.1 + C14 + C14.1 + C14.1.OH + C14.2 + C14.2.OH + C16 + C16.1.OH + C16.2 + C18 + C18.1 + C18.2) |
| Phospholipase A2 Activity (1) | pla2 | (lysoPC.a.C16.0 + lysoPC.a.C16.1 + lysoPC.a.C17.0 + lysoPC.a.C18.0 + lysoPC.a.C18.1 + lysoPC.a.C18.2 + lysoPC.a.C20.3 + lysoPC.a.C20.4) / (PC.aa.C28.1 + PC.aa.C30.0 + PC.aa.C32.0 + PC.aa.C32.1 + PC.aa.C32.2 + PC.aa.C32.3 + PC.aa.C34.1 + PC.aa.C34.2 + PC.aa.C34.3 + PC.aa.C34.4 + PC.aa.C36.1 + PC.aa.C36.2 + PC.aa.C36.3 + PC.aa.C36.4 + PC.aa.C36.5 + PC.aa.C36.6 + PC.aa.C38.0 + PC.aa.C38.1 + PC.aa.C38.3 + PC.aa.C38.4 + PC.aa.C38.5 + PC.aa.C38.6 + PC.aa.C40.2 + PC.aa.C40.3 + PC.aa.C40.4 + PC.aa.C40.5 + PC.aa.C40.6 + PC.aa.C42.0 + PC.aa.C42.1 + PC.aa.C42.2 + PC.aa.C42.4 + PC.aa.C42.5 + PC.aa.C42.6 + PC.ae.C30.0 + PC.ae.C32.1 + PC.ae.C32.2 + PC.ae.C34.0 + PC.ae.C34.1 + PC.ae.C34.2 + PC.ae.C34.3 + PC.ae.C36.0 + PC.ae.C36.1 + PC.ae.C36.2 + PC.ae.C36.3 + PC.ae.C36.4 + PC.ae.C36.5 + PC.ae.C38.0 + PC.ae.C38.1 + PC.ae.C38.2 + PC.ae.C38.3 + PC.ae.C38.4 + PC.ae.C38.5 + PC.ae.C38.6 + PC.ae.C40.1 + PC.ae.C40.2 + PC.ae.C40.3 + PC.ae.C40.4 + PC.ae.C40.5 + PC.ae.C40.6 + PC.ae.C42.1 + PC.ae.C42.2 + PC.ae.C42.3 + PC.ae.C42.4 + PC.ae.C42.5 + PC.ae.C44.3 + PC.ae.C44.4 + PC.ae.C44.5 + PC.ae.C44.6) |
| Sum of Lysophosphatidylcholines | total_lysoPC | lysoPC.a.C16.0 + lysoPC.a.C16.1 + lysoPC.a.C17.0 + lysoPC.a.C18.0 + lysoPC.a.C18.1 + lysoPC.a.C18.2 + lysoPC.a.C20.3 + lysoPC.a.C20.4 |
| Sum of Monounsaturated Fatty Acid Lysophosphatidylcholines | mufa_lysoPC | lysoPC.a.C16.1 + lysoPC.a.C18.1 |
| Sum of Polyunsaturated Fatty Acid Lysophosphatidylcholines | pufa_lysoPC | lysoPC.a.C18.2 + lysoPC.a.C20.3 + lysoPC.a.C20.4 |
| Sum of Saturated Fatty Acid Lysophosphatidylcholines | sfa_lysoPC | lysoPC.a.C16.0 + lysoPC.a.C17.0 + lysoPC.a.C18.0 |
| Ratio of Monounsaturated Fatty Acid Phosphatidylcholines to Saturated Fatty  Acid Phosphatidylcholines | mufaPC_sfaPC | (PC.aa.C28.1 + PC.aa.C32.1 + PC.aa.C34.1 + PC.aa.C36.1 + PC.aa.C38.1 + PC.aa.C42.1 + PC.ae.C32.1 + PC.ae.C34.1 + PC.ae.C36.1 + PC.ae.C38.1 + PC.ae.C40.1 + PC.ae.C42.1) / (PC.aa.C30.0 + PC.aa.C32.0 + PC.aa.C38.0 + PC.aa.C42.0 + PC.ae.C30.0 + PC.ae.C34.0 + PC.ae.C36.0 + PC.ae.C38.0) |
| Ratio of Acyl-Alkyl-Phosphatidylcholines to Diacyl-Phosphatidylcholines | PCae_PCaa | (PC.ae.C30.0 + PC.ae.C32.1 + PC.ae.C32.2 + PC.ae.C34.0 + PC.ae.C34.1 + PC.ae.C34.2 + PC.ae.C34.3 + PC.ae.C36.0 + PC.ae.C36.1 + PC.ae.C36.2 + PC.ae.C36.3 + PC.ae.C36.4 + PC.ae.C36.5 + PC.ae.C38.0 + PC.ae.C38.1 + PC.ae.C38.2 + PC.ae.C38.3 + PC.ae.C38.4 + PC.ae.C38.5 + PC.ae.C38.6 + PC.ae.C40.1 + PC.ae.C40.2 + PC.ae.C40.3 + PC.ae.C40.4 + PC.ae.C40.5 + PC.ae.C40.6 + PC.ae.C42.1 + PC.ae.C42.2 + PC.ae.C42.3 + PC.ae.C42.4 + PC.ae.C42.5 + PC.ae.C44.3 + PC.ae.C44.4 + PC.ae.C44.5 + PC.ae.C44.6) / (PC.aa.C28.1 + PC.aa.C30.0 + PC.aa.C32.0 + PC.aa.C32.1 + PC.aa.C32.2 + PC.aa.C32.3 + PC.aa.C34.1 + PC.aa.C34.2 + PC.aa.C34.3 + PC.aa.C34.4 + PC.aa.C36.1 + PC.aa.C36.2 + PC.aa.C36.3 + PC.aa.C36.4 + PC.aa.C36.5 + PC.aa.C36.6 + PC.aa.C38.0 + PC.aa.C38.1 + PC.aa.C38.3 + PC.aa.C38.4 + PC.aa.C38.5 + PC.aa.C38.6 + PC.aa.C40.2 + PC.aa.C40.3 + PC.aa.C40.4 + PC.aa.C40.5 + PC.aa.C40.6 + PC.aa.C42.0 + PC.aa.C42.1 + PC.aa.C42.2 + PC.aa.C42.4 + PC.aa.C42.5 + PC.aa.C42.6) |
| Ratio of Polyunsaturated Fatty Acid Phosphatidylcholines to Monounsaturated Fatty Acid Phosphatidylcholines | pufaPC_mufaPC | (PC.aa.C32.2 + PC.aa.C32.3 + PC.aa.C34.2 + PC.aa.C34.3 + PC.aa.C34.4 + PC.aa.C36.2 + PC.aa.C36.3 + PC.aa.C36.4 + PC.aa.C36.5 + PC.aa.C36.6 + PC.aa.C38.3 + PC.aa.C38.4 + PC.aa.C38.5 + PC.aa.C38.6 + PC.aa.C40.2 + PC.aa.C40.3 + PC.aa.C40.4 + PC.aa.C40.5 + PC.aa.C40.6 + PC.aa.C42.2 + PC.aa.C42.4 + PC.aa.C42.5 + PC.aa.C42.6 + PC.ae.C32.2 + PC.ae.C34.2 + PC.ae.C34.3 + PC.ae.C36.2 + PC.ae.C36.3 + PC.ae.C36.4 + PC.ae.C36.5 + PC.ae.C38.2 + PC.ae.C38.3 + PC.ae.C38.4 + PC.ae.C38.5 + PC.ae.C38.6 + PC.ae.C40.2 + PC.ae.C40.3 + PC.ae.C40.4 + PC.ae.C40.5 + PC.ae.C40.6 + PC.ae.C42.1 + PC.ae.C42.2 + PC.ae.C42.3 + PC.ae.C42.4 + PC.ae.C42.5 + PC.ae.C44.3 + PC.ae.C44.4 + PC.ae.C44.5 + PC.ae.C44.6) / (PC.aa.C28.1 + PC.aa.C32.1 + PC.aa.C34.1 + PC.aa.C36.1 + PC.aa.C38.1 + PC.aa.C42.1 + PC.ae.C32.1 + PC.ae.C34.1 + PC.ae.C36.1 + PC.ae.C38.1 + PC.ae.C40.1 + PC.ae.C42.1) |
| Ratio of Polyunsaturated Fatty Acid Phosphatidylcholines to Saturated Fatty  Acid Phosphatidylcholines | pufaPC_sfaPC | (PC.aa.C32.2 + PC.aa.C32.3 + PC.aa.C34.2 + PC.aa.C34.3 + PC.aa.C34.4 + PC.aa.C36.2 + PC.aa.C36.3 + PC.aa.C36.4 + PC.aa.C36.5 + PC.aa.C36.6 + PC.aa.C38.3 + PC.aa.C38.4 + PC.aa.C38.5 + PC.aa.C38.6 + PC.aa.C40.2 + PC.aa.C40.3 + PC.aa.C40.4 + PC.aa.C40.5 + PC.aa.C40.6 + PC.aa.C42.2 + PC.aa.C42.4 + PC.aa.C42.5 + PC.aa.C42.6 + PC.ae.C32.2 + PC.ae.C34.2 + PC.ae.C34.3 + PC.ae.C36.2 + PC.ae.C36.3 + PC.ae.C36.4 + PC.ae.C36.5 + PC.ae.C38.2 + PC.ae.C38.3 + PC.ae.C38.4 + PC.ae.C38.5 + PC.ae.C38.6 + PC.ae.C40.2 + PC.ae.C40.3 + PC.ae.C40.4 + PC.ae.C40.5 + PC.ae.C40.6 + PC.ae.C42.1 + PC.ae.C42.2 + PC.ae.C42.3 + PC.ae.C42.4 + PC.ae.C42.5 + PC.ae.C44.3 + PC.ae.C44.4 + PC.ae.C44.5 + PC.ae.C44.6) / (PC.aa.C30.0 + PC.aa.C32.0 + PC.aa.C38.0 + PC.aa.C42.0 + PC.ae.C30.0 + PC.ae.C34.0 + PC.ae.C36.0 + PC.ae.C38.0) |
| Sum of Monounsaturated Fatty Acid Phosphatidylcholines | total_mufaPCax | PC.aa.C28.1 + PC.aa.C32.1 + PC.aa.C34.1 + PC.aa.C36.1 + PC.aa.C38.1 + PC.aa.C42.1 + PC.ae.C32.1 + PC.ae.C34.1 + PC.ae.C36.1 + PC.ae.C38.1 + PC.ae.C40.1 + PC.ae.C42.1 |
| Sum of Phosphatidylcholines | total_PCax | PC.aa.C28.1 + PC.aa.C30.0 + PC.aa.C32.0 + PC.aa.C32.1 + PC.aa.C32.2 + PC.aa.C32.3 + PC.aa.C34.1 + PC.aa.C34.2 + PC.aa.C34.3 + PC.aa.C34.4 + PC.aa.C36.1 + PC.aa.C36.2 + PC.aa.C36.3 + PC.aa.C36.4 + PC.aa.C36.5 + PC.aa.C36.6 + PC.aa.C38.0 + PC.aa.C38.1 + PC.aa.C38.3 + PC.aa.C38.4 + PC.aa.C38.5 + PC.aa.C38.6 + PC.aa.C40.2 + PC.aa.C40.3 + PC.aa.C40.4 + PC.aa.C40.5 + PC.aa.C40.6 + PC.aa.C42.0 + PC.aa.C42.1 + PC.aa.C42.2 + PC.aa.C42.4 + PC.aa.C42.5 + PC.aa.C42.6 + PC.ae.C30.0 + PC.ae.C32.1 + PC.ae.C32.2 + PC.ae.C34.0 + PC.ae.C34.1 + PC.ae.C34.2 + PC.ae.C34.3 + PC.ae.C36.0 + PC.ae.C36.1 + PC.ae.C36.2 + PC.ae.C36.3 + PC.ae.C36.4 + PC.ae.C36.5 + PC.ae.C38.0 + PC.ae.C38.1 + PC.ae.C38.2 + PC.ae.C38.3 + PC.ae.C38.4 + PC.ae.C38.5 + PC.ae.C38.6 + PC.ae.C40.1 + PC.ae.C40.2 + PC.ae.C40.3 + PC.ae.C40.4 + PC.ae.C40.5 + PC.ae.C40.6 + PC.ae.C42.1 + PC.ae.C42.2 + PC.ae.C42.3 + PC.ae.C42.4 + PC.ae.C42.5 + PC.ae.C44.3 + PC.ae.C44.4 + PC.ae.C44.5 + PC.ae.C44.6 |
| Sum of Diacyl-Phosphatidylcholines | total_PCaa | PC.aa.C28.1 + PC.aa.C30.0 + PC.aa.C32.0 + PC.aa.C32.1 + PC.aa.C32.2 + PC.aa.C32.3 + PC.aa.C34.1 + PC.aa.C34.2 + PC.aa.C34.3 + PC.aa.C34.4 + PC.aa.C36.1 + PC.aa.C36.2 + PC.aa.C36.3 + PC.aa.C36.4 + PC.aa.C36.5 + PC.aa.C36.6 + PC.aa.C38.0 + PC.aa.C38.1 + PC.aa.C38.3 + PC.aa.C38.4 + PC.aa.C38.5 + PC.aa.C38.6 + PC.aa.C40.2 + PC.aa.C40.3 + PC.aa.C40.4 + PC.aa.C40.5 + PC.aa.C40.6 + PC.aa.C42.0 + PC.aa.C42.1 + PC.aa.C42.2 + PC.aa.C42.4 + PC.aa.C42.5 + PC.aa.C42.6 |
| Sum of Acyl-Alkyl-Phosphatidylcholines | total_PCae | PC.ae.C30.0 + PC.ae.C32.1 + PC.ae.C32.2 + PC.ae.C34.0 + PC.ae.C34.1 + PC.ae.C34.2 + PC.ae.C34.3 + PC.ae.C36.0 + PC.ae.C36.1 + PC.ae.C36.2 + PC.ae.C36.3 + PC.ae.C36.4 + PC.ae.C36.5 + PC.ae.C38.0 + PC.ae.C38.1 + PC.ae.C38.2 + PC.ae.C38.3 + PC.ae.C38.4 + PC.ae.C38.5 + PC.ae.C38.6 + PC.ae.C40.1 + PC.ae.C40.2 + PC.ae.C40.3 + PC.ae.C40.4 + PC.ae.C40.5 + PC.ae.C40.6 + PC.ae.C42.1 + PC.ae.C42.2 + PC.ae.C42.3 + PC.ae.C42.4 + PC.ae.C42.5 + PC.ae.C44.3 + PC.ae.C44.4 + PC.ae.C44.5 + PC.ae.C44.6 |
| Sum of Polyunsaturated Fatty Acid Phosphatidylcholines | total_pufaPCax | PC.aa.C32.2 + PC.aa.C32.3 + PC.aa.C34.2 + PC.aa.C34.3 + PC.aa.C34.4 + PC.aa.C36.2 + PC.aa.C36.3 + PC.aa.C36.4 + PC.aa.C36.5 + PC.aa.C36.6 + PC.aa.C38.3 + PC.aa.C38.4 + PC.aa.C38.5 + PC.aa.C38.6 + PC.aa.C40.2 + PC.aa.C40.3 + PC.aa.C40.4 + PC.aa.C40.5 + PC.aa.C40.6 + PC.aa.C42.2 + PC.aa.C42.4 + PC.aa.C42.5 + PC.aa.C42.6 + PC.ae.C32.2 + PC.ae.C34.2 + PC.ae.C34.3 + PC.ae.C36.2 + PC.ae.C36.3 + PC.ae.C36.4 + PC.ae.C36.5 + PC.ae.C38.2 + PC.ae.C38.3 + PC.ae.C38.4 + PC.ae.C38.5 + PC.ae.C38.6 + PC.ae.C40.2 + PC.ae.C40.3 + PC.ae.C40.4 + PC.ae.C40.5 + PC.ae.C40.6 + PC.ae.C42.1 + PC.ae.C42.2 + PC.ae.C42.3 + PC.ae.C42.4 + PC.ae.C42.5 + PC.ae.C44.3 + PC.ae.C44.4 + PC.ae.C44.5 + PC.ae.C44.6 |
| Sum of Saturated Fatty Acid Phosphatidylcholines | total_sfaPCax | PC.aa.C30.0 + PC.aa.C32.0 + PC.aa.C38.0 + PC.aa.C42.0 + PC.ae.C30.0 + PC.ae.C34.0 + PC.ae.C36.0 + PC.ae.C38.0 |
| Ratio of Hydroxylated Sphingomyelins to  Non-Hydroxylated Sphingomyelins | SM.OH_SMnonOH | (SM..OH..C14.1 + SM..OH..C16.1 + SM..OH..C22.1 + SM..OH..C22.2 + SM..OH..C24.1) / (SM.C16.0 + SM.C16.1 + SM.C18.0 + SM.C18.1 + SM.C20.2 + SM.C24.0 + SM.C24.1) |
| Ratio of Sphingomyelins to Phosphatidylcholines | SM_PC | (SM..OH..C14.1 + SM..OH..C16.1 + SM..OH..C22.1 + SM..OH..C22.2 + SM..OH..C24.1 + SM.C16.0 + SM.C16.1 + SM.C18.0 + SM.C18.1 + SM.C20.2 + SM.C24.0 + SM.C24.1) / (PC.aa.C28.1 + PC.aa.C30.0 + PC.aa.C32.0 + PC.aa.C32.1 + PC.aa.C32.2 + PC.aa.C32.3 + PC.aa.C34.1 + PC.aa.C34.2 + PC.aa.C34.3 + PC.aa.C34.4 + PC.aa.C36.1 + PC.aa.C36.2 + PC.aa.C36.3 + PC.aa.C36.4 + PC.aa.C36.5 + PC.aa.C36.6 + PC.aa.C38.0 + PC.aa.C38.1 + PC.aa.C38.3 + PC.aa.C38.4 + PC.aa.C38.5 + PC.aa.C38.6 + PC.aa.C40.2 + PC.aa.C40.3 + PC.aa.C40.4 + PC.aa.C40.5 + PC.aa.C40.6 + PC.aa.C42.0 + PC.aa.C42.1 + PC.aa.C42.2 + PC.aa.C42.4 + PC.aa.C42.5 + PC.aa.C42.6 + PC.ae.C30.0 + PC.ae.C32.1 + PC.ae.C32.2 + PC.ae.C34.0 + PC.ae.C34.1 + PC.ae.C34.2 + PC.ae.C34.3 + PC.ae.C36.0 + PC.ae.C36.1 + PC.ae.C36.2 + PC.ae.C36.3 + PC.ae.C36.4 + PC.ae.C36.5 + PC.ae.C38.0 + PC.ae.C38.1 + PC.ae.C38.2 + PC.ae.C38.3 + PC.ae.C38.4 + PC.ae.C38.5 + PC.ae.C38.6 + PC.ae.C40.1 + PC.ae.C40.2 + PC.ae.C40.3 + PC.ae.C40.4 + PC.ae.C40.5 + PC.ae.C40.6 + PC.ae.C42.1 + PC.ae.C42.2 + PC.ae.C42.3 + PC.ae.C42.4 + PC.ae.C42.5 + PC.ae.C44.3 + PC.ae.C44.4 + PC.ae.C44.5 + PC.ae.C44.6) |
| Sum of Non-Hydroxylated Sphingomyelins | total_SM_nonOH | SM.C16.0 + SM.C16.1 + SM.C18.0 + SM.C18.1 + SM.C20.2 + SM.C24.0 + SM.C24.1 |
| Sum of Hydroxylated Sphingomyelins | total_SM.OH | SM..OH..C14.1 + SM..OH..C16.1 + SM..OH..C22.1 + SM..OH..C22.2 + SM..OH..C24.1 |
| Sum of Sphingomyelins | total_SM | SM..OH..C14.1 + SM..OH..C16.1 + SM..OH..C22.1 + SM..OH..C22.2 + SM..OH..C24.1 + SM.C16.0 + SM.C16.1 + SM.C18.0 + SM.C18.1 + SM.C20.2 + SM.C24.0 + SM.C24.1 |

**Supplementary Table 4** Bootstrapped linear regression (5000 resamples) of subphenotype and metabolites. Subphenotype I was used as reference.

| **Metabolite** | **Group** | **Exposure** | **Estimate** | **Low 95% CI** | **High 95% CI** | **p-value** | **FDR p-value** |
| --- | --- | --- | --- | --- | --- | --- | --- |
| C0 | subphenotype | subphenotype IV | 0.75 | 0.34 | 1.1 | < 0.001 | 0.011 |
| C5 | subphenotype | subphenotype IV | 0.63 | 0.29 | 1.01 | < 0.001 | 0.011 |
| Ala | subphenotype | subphenotype IV | 0.74 | 0.3 | 1.16 | < 0.001 | 0.017 |
| Arg | subphenotype | subphenotype V | -0.77 | -1.24 | -0.31 | 0.0024 | 0.038 |
| Glu | subphenotype | subphenotype III | 0.69 | 0.3 | 1.08 | < 0.001 | 0.011 |
| Glu | subphenotype | subphenotype IV | 0.86 | 0.52 | 1.21 | < 0.001 | < 0.001 |
| Glu | subphenotype | subphenotype V | 0.85 | 0.42 | 1.23 | < 0.001 | < 0.001 |
| Gly | subphenotype | subphenotype V | -0.66 | -1.07 | -0.22 | 0.0032 | 0.048 |
| Phe | subphenotype | subphenotype IV | 0.72 | 0.36 | 1.09 | < 0.001 | < 0.001 |
| Tyr | subphenotype | subphenotype IV | 0.82 | 0.46 | 1.22 | < 0.001 | < 0.001 |
| Kynurenine | subphenotype | subphenotype IV | 0.69 | 0.36 | 1.08 | < 0.001 | 0.011 |
| lysoPC.a.C17.0 | subphenotype | subphenotype III | -0.66 | -1.08 | -0.26 | 0.0012 | 0.023 |
| lysoPC.a.C17.0 | subphenotype | subphenotype IV | -0.88 | -1.28 | -0.51 | < 0.001 | < 0.001 |
| lysoPC.a.C17.0 | subphenotype | subphenotype V | -0.9 | -1.37 | -0.51 | < 0.001 | < 0.001 |
| lysoPC.a.C18.1 | subphenotype | subphenotype III | -0.66 | -1.07 | -0.27 | < 0.001 | 0.017 |
| lysoPC.a.C18.2 | subphenotype | subphenotype II | -0.46 | -0.75 | -0.18 | < 0.001 | 0.017 |
| lysoPC.a.C18.2 | subphenotype | subphenotype III | -0.85 | -1.25 | -0.45 | < 0.001 | 0.011 |
| lysoPC.a.C18.2 | subphenotype | subphenotype IV | -0.73 | -1.11 | -0.37 | < 0.001 | 0.017 |
| lysoPC.a.C18.2 | subphenotype | subphenotype V | -0.9 | -1.34 | -0.45 | < 0.001 | < 0.001 |
| PC.aa.C32.1 | subphenotype | subphenotype IV | 0.77 | 0.39 | 1.15 | < 0.001 | < 0.001 |
| PC.aa.C32.1 | subphenotype | subphenotype V | 0.72 | 0.26 | 1.15 | 0.002 | 0.033 |
| PC.aa.C32.2 | subphenotype | subphenotype II | 0.54 | 0.24 | 0.85 | 0.0016 | 0.029 |
| PC.aa.C32.2 | subphenotype | subphenotype V | 0.62 | 0.21 | 1.03 | 0.0032 | 0.048 |
| PC.aa.C32.3 | subphenotype | subphenotype II | 0.53 | 0.24 | 0.81 | < 0.001 | 0.011 |
| PC.aa.C34.4 | subphenotype | subphenotype II | 0.61 | 0.3 | 0.92 | < 0.001 | < 0.001 |
| PC.aa.C34.4 | subphenotype | subphenotype V | 0.84 | 0.4 | 1.29 | < 0.001 | < 0.001 |
| PC.aa.C38.3 | subphenotype | subphenotype IV | 0.81 | 0.42 | 1.13 | < 0.001 | < 0.001 |
| PC.aa.C38.3 | subphenotype | subphenotype V | 0.67 | 0.28 | 1.05 | < 0.001 | 0.011 |
| PC.aa.C40.5 | subphenotype | subphenotype II | 0.51 | 0.22 | 0.83 | < 0.001 | 0.017 |
| PC.aa.C40.5 | subphenotype | subphenotype IV | 0.79 | 0.43 | 1.17 | < 0.001 | < 0.001 |
| PC.aa.C40.5 | subphenotype | subphenotype V | 0.65 | 0.22 | 1.09 | 0.0024 | 0.038 |
| SM..OH..C22.1 | subphenotype | subphenotype II | 0.5 | 0.19 | 0.8 | 0.0012 | 0.023 |
| SM.C18.1 | subphenotype | subphenotype II | 0.49 | 0.22 | 0.78 | < 0.001 | < 0.001 |
| SM.C20.2 | subphenotype | subphenotype II | 0.49 | 0.19 | 0.79 | 0.0016 | 0.029 |
| SM.C20.2 | subphenotype | subphenotype III | 0.59 | 0.21 | 0.98 | 0.0012 | 0.023 |
| SM.C24.0 | subphenotype | subphenotype II | 0.66 | 0.35 | 0.97 | < 0.001 | < 0.001 |
| SM.C24.0 | subphenotype | subphenotype III | 0.67 | 0.24 | 1.14 | 0.002 | 0.033 |
| H1 | subphenotype | subphenotype II | 0.42 | 0.17 | 0.72 | < 0.001 | 0.017 |
| H1 | subphenotype | subphenotype IV | 0.59 | 0.24 | 0.98 | 0.002 | 0.033 |
| pro_cit | subphenotype | subphenotype II | 0.56 | 0.26 | 0.86 | < 0.001 | 0.007 |
| aaa | subphenotype | subphenotype IV | 0.65 | 0.26 | 1.06 | 0.0028 | 0.032 |
| orn_arg | subphenotype | subphenotype V | 0.71 | 0.28 | 1.16 | 0.0016 | 0.023 |
| omega_ox | subphenotype | subphenotype III | -0.8 | -1.24 | -0.35 | < 0.001 | < 0.001 |
| omega_ox | subphenotype | subphenotype IV | -0.81 | -1.21 | -0.43 | < 0.001 | < 0.001 |
| total_medAC | subphenotype | subphenotype II | 0.47 | 0.17 | 0.8 | 0.0028 | 0.032 |
| pla2 | subphenotype | subphenotype II | -0.63 | -0.89 | -0.36 | < 0.001 | < 0.001 |
| pla2 | subphenotype | subphenotype III | -0.86 | -1.24 | -0.51 | < 0.001 | < 0.001 |
| pla2 | subphenotype | subphenotype IV | -0.82 | -1.17 | -0.43 | < 0.001 | < 0.001 |
| pla2 | subphenotype | subphenotype V | -0.96 | -1.42 | -0.5 | < 0.001 | < 0.001 |
| total_lysoPC | subphenotype | subphenotype III | -0.62 | -1.03 | -0.21 | 0.004 | 0.04 |
| mufa_lysoPC | subphenotype | subphenotype III | -0.59 | -0.99 | -0.19 | 0.0036 | 0.038 |
| pufa_lysoPC | subphenotype | subphenotype III | -0.84 | -1.26 | -0.41 | < 0.001 | 0.007 |
| pufa_lysoPC | subphenotype | subphenotype IV | -0.67 | -1.06 | -0.3 | < 0.001 | 0.007 |
| pufa_lysoPC | subphenotype | subphenotype V | -0.81 | -1.25 | -0.36 | < 0.001 | 0.013 |
| PCae_PCaa | subphenotype | subphenotype V | -0.63 | -1.03 | -0.26 | 0.002 | 0.027 |

References

1. Rospleszcz S, Lorbeer R, Storz C, Schlett CL, Meisinger C, Thorand B, Rathmann W, Bamberg F, Lieb W, Peters A: **Association of longitudinal risk profile trajectory clusters with adipose tissue depots measured by magnetic resonance imaging**. *Scientific reports* 2019, **9**:16972.

2. Bertheau RC, Lorbeer R, Nattenmüller J, Wintermeyer E, Machann J, Linkohr B, Peters A, Bamberg F, Schlett CL: **Bone marrow fat fraction assessment in regard to physical activity: KORA FF4-3-T MR imaging in a population-based cohort**. *European radiology* 2020, **30**:3417-3428.

3. Kiefer LS, Fabian J, Lorbeer R, Machann J, Storz C, Kraus MS, Wintermeyer E, Schlett C, Roemer F, Nikolaou K, Peters A, Bamberg F: **Inter- and intra-observer variability of an anatomical landmark-based, manual segmentation method by MRI for the assessment of skeletal muscle fat content and area in subjects from the general population**. *The British journal of radiology* 2018, **91**:20180019.

4. Kiefer LS, Fabian J, Rospleszcz S, Lorbeer R, Machann J, Kraus MS, Roemer F, Rathmann W, Meisinger C, Heier M, Nikolaou K, Peters A, Storz C, Diallo TD, Schlett CL, Bamberg F: **Distribution patterns of intramyocellular and extramyocellular fat by magnetic resonance imaging in subjects with diabetes, prediabetes and normoglycaemic controls**. *Diabetes, obesity & metabolism* 2021, **23**:1868-1878.

5. Rado SD, Lorbeer R, Gatidis S, Machann J, Storz C, Nikolaou K, Rathmann W, Hoffmann U, Peters A, Bamberg F, Schlett CL: **MRI-based assessment and characterization of epicardial and paracardial fat depots in the context of impaired glucose metabolism and subclinical left-ventricular alterations**. *The British journal of radiology* 2019, **92**:20180562.

6. Grune E, Nattenmüller J, Kiefer LS, Machann J, Peters A, Bamberg F, Schlett CL, Rospleszcz S: **Subphenotypes of body composition and their association with cardiometabolic risk - Magnetic resonance imaging in a population-based sample**. *Metabolism: clinical and experimental* 2025, **164**:156130.

7. Notohamiprodjo M, Goepfert M, Will S, Lorbeer R, Schick F, Rathmann W, Martirosian P, Peters A, Müller-Peltzer K, Helck A, Rospleszcz S, Bamberg F: **Renal and renal sinus fat volumes as quantified by magnetic resonance imaging in subjects with prediabetes, diabetes, and normal glucose tolerance**. *PloS one* 2020, **15**:e0216635.

8. Kyle UG, Genton L, Karsegard L, Slosman DO, Pichard C: **Single prediction equation for bioelectrical impedance analysis in adults aged 20--94 years**. *Nutrition (Burbank, Los Angeles County, Calif.)* 2001, **17**:248-253.

9. Huemer M-T, Bauer A, Petrera A, Scholz M, Hauck SM, Drey M, Peters A, Thorand B: **Proteomic profiling of low muscle and high fat mass: a machine learning approach in the KORA S4/FF4 study**. *Journal of cachexia, sarcopenia and muscle* 2021, **12**:1011-1023.

10. Maushagen J, Nattenmüller J, Krüchten R von, Thorand B, Peters A, Rathmann W, Adamski J, Schlett CL, Bamberg F, Wang-Sattler R, Rospleszcz S: **Serum metabolites characterize hepatic phenotypes and reveal shared pathways: results from population-based imaging**. *Molecular medicine (Cambridge, Mass.)* 2025, **31**:260.
